# Genome-Wide Association Mapping and Gene Expression Analyses Reveal Genetic Mechanisms of Disease Resistance Variations in *Cynoglossus semilaevis*

**DOI:** 10.3389/fgene.2019.01167

**Published:** 2019-11-20

**Authors:** Qian Zhou, Zhencheng Su, Yangzhen Li, Yang Liu, Lei Wang, Sheng Lu, Shuanyan Wang, Tian Gan, Feng Liu, Xun Zhou, Min Wei, Guangjian Liu, Songlin Chen

**Affiliations:** ^1^Yellow Sea Fisheries Research Institute, Chinese Academy of Fishery Sciences/Key Laboratory for Sustainable Development of Marine Fisheries, Ministry of Agriculture, Qingdao, China; ^2^Laboratory for Marine Fisheries Science and Food Production Processes, Pilot National Laboratory for Marine Science and Technology, Qingdao, China; ^3^Key Laboratory for Marine Fishery Biotechnology and Genetic Breeding, Qingdao, China; ^4^Novogene Bioinformatics Technology Co., Ltd, Beijing, China

**Keywords:** genome re-sequencing, genome-wide association study, Fst and nucleotide diversity filtration, disease resistance, *Cynoglossus semilaevis*

## Abstract

The sustainable development of aquaculture has been impeded by infectious diseases worldwide. However, the genomic architecture and the genetic basis underlying the disease resistance remain poorly understood, which severely hampers both the understanding of the evolution of fish disease resistance traits and the prevention of these diseases in the aquaculture community. *Cynoglossus semilaevis* is a representative and commercially-important flatfish species. Here we combined genome-wide association study and Fst and nucleotide diversity filtration to identify loci important for the disease resistance. Based on 1,016,774 single-nucleotide polymorphisms (SNPs) identified from 650 Gb genome resequencing data of 505 individuals, we detected 33 SNPs significantly associated with disease resistance and 79 candidate regions after filtration steps. Both the allele frequencies and genotype frequencies of the associated loci were significantly different between the resistant and susceptible fish, suggesting a role in the genetic basis of disease resistance. The SNP with strongest association with disease resistance was located in Chr 17, at 145 bp upstream of *fblx19* gene, and overlapped with the major quantitative trait locus previously identified. Several genes, such as *plekha7*, *nucb2*, and *fgfr2*, were also identified to potentially play roles in the disease resistance. Furthermore, the expression of some associating genes were likely under epigenetic regulations between the bacterial resistant and susceptible families. These results provide insights into the mechanism that enable variation of disease resistance to bacterial pathogen infection. The identified polymorphisms and genes are valuable targets and molecular resources for disease resistance and other traits, and for advanced breeding practice for superior germplasm in fish aquaculture.

## Introduction

Aquaculture has been playing an important role in food security and rural economic development in both developing and developed countries. Recently, the development of aquaculture industry has been heavily arrested by some prevalent problems worldwide, such as degeneration of breeding germplasm and occurrences of diseases. Breeding of superior varieties is a highly efficient and environment-friendly solution to improve the quality and sustainability of the aquatic farming. The detection of genome-wide genetic diversity and the identification of genes contributing to phenotypic improvement play essential roles in achieving this goal. In aquaculture species, genomic resources have been explored for economically important traits like growth, sex determination, and disease resistance (reviewed by Robledo et al., 2018). Finding the protective or deleterious alleles and genes, and selective breeding on individuals with corresponding haplotypes would increase the health and welfare for the aquaculture.

Frequent outbreaks of diseases have caused tremendous mortalities and production loss in fish farming. In the last decade, a number of studies were conducted to elucidate the genomic basis of the responses to bacterial infections in fish. RNA-Seq identified a number of key genes regarding to the bacterial infections in catfish (Li et al., 2012; Sun et al., 2012), sea bass (*Lateolabrax japonicas*; Xiang et al., 2010), and Chinese tongue sole (*Cynoglossus semilaevis,* Zhang et al., 2015). MicroRNA profiles were also characterized to play roles in host–pathogen interactions in various fish, such as *C. semilaevis* (Sha et al., 2014), common carp (Reichert et al., 2019), Nile tilapia (Gao et al., 2019), and rainbow trout (*Oncorhynchus mykiss*, Cao et al., 2018). Quantitative trait locus (QTL) mappings were previously applied to locate the regions associated with disease resistance in aquaculture fish, such as Atlantic salmon (*Salmo salar*) (Houston et al., 2008; Gheyas et al, 2010; Gonen et al., 2015), rainbow trout (Baerwald et al., 2011; Palti et al., 2015), Japanese flounder (*Paralichthys olivaceus*, Shao et al., 2015), and *C. semilaevis* (Dai et al., 2017). The application of genome-wide association study (GWAS) revealed the polygenic architecture of the host resistance to pathogens, and allowed detection of the single-nucleotide polymorphisms (SNPs) and genes associated with disease resistance in many fish, such as catfish (Xin et al., 2015), Atlantic salmon (Correa et al., 2015; Robledo et al., 2018), sea bream (*Sparus aurata*; Palaiokostas et al., 2016), and rainbow trout (Campbell et al., 2014; Vallejo et al., 2016). Recently, major histocompatibility complex IIA polymorphisms have been found to be associated with disease resistance in Nile tilapia (El-Magd et al., 2019). In flatfish species, QTLs for resistance to *Aeromonas salmonicida* were identified in turbot (Rodríguez-Ramilo et al., 2011). A major locus on G15 (marker Poli.9-8TUF) for resistance to lymphocystis disease has been reported and successfully applied as a selection breeding marker in Japanese flounder (Fuji et al., 2006; Fuji et al., 2007). Furthermore, the toll-like receptor 2 has been identified as a candidate gene for resistance to lymphocystis disease, which was mapped with the Poli.9-8TUF marker (Hwang et al., 2011). In addition, some alleles in major histocompatibility complex class IIB gene that associated with resistance against *Vibrio anguillarum* have also been found in this species (Xu et al., 2008). As a quantitative trait, disease resistance generally has a complex genetic architecture, in which many genes are involved and large numbers of loci each explain very little genetic variation.

The Chinese tongue sole, *C. semilaevis*, a representative marine flatfish species in China, has a high commercial value in aquaculture. Recently, the farming industry of *C. semilaevis* is undergoing a dramatical decline due to devastating diseases. From 2005, we have conducted continuous selective breeding for *C. semilaevis* by family construction and pathogen challenges using the bacterium *Vibrio harveyi*, which caused prevalent and high mortality across farms. We found that even in strictly controlled and highly consistent cultivating conditions, fishes from different families exhibited distinct phenotypic difference in the disease resistance/susceptibility (Chen et al., 2010; Li et al., 2019). Dissecting the genetics and genomic mechanisms of the disease resistance require detections of genomic signatures of associations between genotype and phenotype. The availability of the whole genome sequence of *C. semilaevis* (Chen et al., 2014) provides the foundation for profound genetic studies.

Here we identify genetic loci significantly associated with disease resistance, using genome resequencing data of 505 individuals of *C. semilaevis*. Our findings provide insights into the evolution and genomic mechanisms underlying the disease resistance, as well as genetic targets for precise genetic engineering and advanced breeding practice. These results hold a great potential for the prevention of the infectious disease, the main cause of death in fish aquaculture, and for the breeding of elite germplasm using genomic selection and other technologies.

## Materials and Methods

### Fish Origin and Whole Genome Resequencing

Fish were collected from two farming factories located in Laizhou (LZ) and Haiyang (HY), China, respectively. We established the initial brood stocks in 2005 using wild individuals and performed breeding programs by crossing the selected *V. harveyi* resistant males with fast growing or wild females. After *V. harveyi* challenge, the survival rates of each family were calculated and the family having a survival rate >80% were considered as high resistant family. Till now, the culturing stocks have undergone approximately three to four generations of selection for resistance to the *V. harveyi* infection. In the 2014 breeding year, we constructed 106 families using 230 parents, and tagged all the families with fluorescent markers to record their pedigree information. All the F1 hybrids were maintained in the same conditions and 30 ± 5 individuals at 4–5 months posthatch (mph), with body weights of 10.76 ± 3.69 g and body lengths of 12.35 ± 1.63 cm, were randomly sampled from each of the 106 families. Thus, more than 3,000 fish were collected for infection experiment. We conducted *V. harveyi* challenge tests by intraperitoneal injection with a medial lethal dose (LD_50_) of 5 × 10^5^ colony-forming units/gram fish, which was determined using the method similar to that reported by Xiong et al., 2017. Mortalities were collected daily for 10 days after injection, and the results showed that the daily mortality rate returned to baseline levels at ∼200 h postinjection (hpi) (**Figure S1**). Therefore, we monitored the mortality for 300 h and collected the fish that died within 72 hpi and survived 300 hpi. Finally, a total of 505 individuals from 105 families, including 389 surviving ones from 88 families and 116 dead ones from 77 families were subjected to genome-resequencing and further analyses. The sex of these individuals were identified using a sex specific amplified fragment-length polymorphism marker (Chen et al., 2007).

Briefly, we extracted the genomic DNA from the fin tissue of each fish using DNeasy Blood & Tissue Kit (Qiagen). Pair-ended libraries were constructed following the standard protocol (Illumina, USA) with an insert distance of 300 bp. Paired reads with a read length of 2 × 100 bp were generated using the Illumina HiSeq2000 platform. We filtered the raw reads using QC-Chain (Zhou et al., 2013), and the adapter sequences, low quality reads, and duplicated reads were removed. Consequently, 1.35–1.8 Gb high quality data were remained for each fish, with an average sequencing depth of 3.0.

### SNP Calling and Annotation

We aligned the high quality reads to the reference genome of *C. semilaevis* (NCBI Accession No. GCA_000523025.1) using Burrows–Wheeler aligner with default settings (Li and Durbin, 2009). The variants calling was performed with SAMtools (v0.1.19, Li et al., 2009) where reads with mapping quality <20 were discarded. Then, we removed the SNPs that had an overall quality score ≤20 and base quality score ≤30. Furthermore, we filtered the SNPs with call rate ≤95%, minor allele frequency (MAF) ≤1% and missing rate ≥10%. In addition, the minimal coverage for SNP calling was 3. We checked the Hardy–Weinberg equilibrium (HWE) using VCFtools (v0.1.14, http://vcftools.sourceforge.net/) with -hwe option. The SNPs departed from HWE with a significance *p*-value < 0.05 were excluded. Then, we used ANNOVAR (Wang et al., 2010) to assign SNP effects according to the annotated gene model. SNPs were grouped into exon, intron, 5′-untranslated region and 3′-untranslated region, upstream and downstream regions (within 1 kb region from the transcription start or stop site), and intergenic regions. SNPs in exonic region were further categorized into synonymous (causing no amino acid changes) or nonsynonymous (causing amino acid changes, stop gain or stop loss) ones.

### Population Structure and Phylogenetic Analyses

To reveal the phylogenetic relationship of the 505 fish from a genome-wide view, we used TreeBest (v1.9.2) (http://treesoft.sourceforge.net/treebest.shtml) to construct a neighbor-joining tree using the filtered SNP set with a bootstrap value of 1,000. The kinship relatedness between all the individuals were calculated. The software Treeview was used for visualizing the phylogenetic tree (http://taxonomy.zoology.gla.ac.uk/rod/treeview.html)^1^. Moreover, we performed principal-component analyses to assess the population structure using GCTA (http://cnsgenomics.com/software/gcta/) and the first two dimensional coordinates were plotted. Linkage disequilibrium (LD) level was measured with the correlation coefficient values (r^2^) between two loci using Haploview (v4.2) software (Barrett et al., 2005). The parameters were set as: -dprime, -minMAF 0.1, -memory 2000, -maxdistance 500.

### GWAS for Disease Resistance

The phenotypes of disease resistance were defined according to the fish responses to the *V. harveyi* infection: we recorded the trait as 0 if the fish died within 72 hpi (DIE) and 1 if the fish survived (SUR) until the end of challenging experiments. We performed single SNP-GWAS using PLINK (v1.07) (Purcell et al., 2007), with the population structure as a fixed effect, and the kinship relatedness matrix of all individuals as a random effect. Significance threshold was calculated using a *p-value* correction where significance was defined as 0.05 divided by number of independently segregating SNPs. The Manhattan plot was generated in R. The allele frequencies were calculated using in-house Python scripts. The identified significantly associated SNPs were tested for pairwise independence using a Fisher's exact test (*p* < 0.05) to group in the same haplotype using Haploview (v4.2). The proportion of phenotypic variance in disease resistance explained by the variants was estimated using TASSEL (Bradbury et al., 2008).

### Fst and Nucleotide Diversity Estimation

To further filter the GWAS identified candidate SNPs, we estimated the fixation index (*Fst*) and the nucleotide diversity (*θπ*) throughout the whole genome (requiring at least 95% accessibility) between the SUR and DIE groups, with the program VCFtools (v0.1.14). We used a 40 kb nonoverlapping window, with a step size of 20 kb, to screen the whole genome and retained the windows containing more than 20 SNPs for further analyses. The *θπ* ratio of *θπ* *_DIE_* */θπ* *_SUR_* was calculated to represent the difference of nucleotide diversity between the SUR and DIE groups. The windows with the top 5% highest values of *Fst* and log_2_(*θπ* ratio) were considered as candidate regions. Adjacent candidate regions within 200 kb were merged into a single candidate region. In addition, we simulated changes in allele frequency across multiple generations to evaluate the influence of genetic drift. We selected a population Ne of 200, a similar value to our initial breeding population of *C. semilaevis*, and carried out simulation analyses using Allele Simulator (Shaffer and Rogan, 2015, http://popgensimulator.pitt.edu/graphs/allele). Changes in allele frequency were calculated over 25 generations in 20 times simulations. T-tests were used to test the significant difference in the allele frequencies in the first and the other generations.

### Gene Expression and Methylation

To analyze the expressions of the identified genes, we sampled 12 fish from two *V. harveyi*-susceptible (VS) (survival rate < 20%) and two resistant (VR) families (survival rate > 80%) that were previously constructed by our lab (three individuals from each family; at 4–5 mph). We collected three immune tissues (liver, spleen and gill) and performed quantitative real-time PCR (qPCR) using the 7500 Real-Time PCR System (Applied Biosystems USA), with *β-actin* gene as the internal control (**Table S1**). Each reaction was performed in a final volume of 20 µl containing 1 × SYBR Premix Ex Taq (Takara), 200 nM each primer, 1 × ROX Reference Dye II (Takara), and 1 µl of the complementary DNA sample. Three replicates were used in the qPCR reactions. The relative expression was analyzed with the 2^−∆∆Ct^ method and the statistical analyses was performed with SPSS 18.0 software (http://www-01.ibm.com/software/analytics/spss/). A t-test *p* < 0.05 and *p* < 0.01 was deemed to indicate a significant and extremely significant difference, respectively. In addition, the genotypes of selected genes of the 12 individuals were obtained using PCR and Sanger sequencing (**Table S2**).

Furthermore, to determine the epigenetic regulation of these genes, we pooled immune tissues, including liver, spleen and kidney and isolated the DNA from the VS and VR families (three individuals per family), and performed the bisulfite conversion and bisulfite sequencing (BS-Seq). The bisulfite conversion of sample DNA was carried out using a modified NH_4_HSO_3_-based protocol (Hayatsu et al., 2006). The pair-ended library construction and sequencing were carried out using Illumina Hiseq 2000 platform. We also mixed 25 ng cl857 Sam7 Lambda DNA in each sample as a conversion quality control for each library. The BS-Seq and differential methylation analyses were performed as previously described (Shao et al., 2014).

## Results

### Whole-Genome Resequencing and SNP Calling

By the genome resequencing of the 505 fish, we generated a total of 650 Gb high quality data, with an average genome coverage of 74.22%. The sequencing reads were aligned to the reference genome and the average mapping rate was 95.44% (**Table S3**). After quality filtration from the initially identified 5,471,595 putative SNPs, we obtained 1,016,774 SNPs with an approximate density of 2.16 SNPs/kb in the genome of *C. semilaevis*. We validated 62 SNPs using Sanger sequencing and the accuracy of SNP calling was >92%. More than 94,562 SNPs were located in exonic regions of 18,535 genes, including 32,117 nonsynonymous, 62,247 synonymous, 182 stop gain (causing premature stop codons), 16 stop loss (causing elongated transcripts), and 207 splicing SNPs (within 2 bp of a splicing junction). In addition, 396,712 SNPs were located in intronic regions, 2,181 were located in upstream or downstream regions, and the remaining 494,168 SNPs were located in intergenic regions (**Table 1**).

**Table 1:** Summary of the single-nucleotide polymorphisms (SNPs) in *Cynoglossus semilaevis*.

Category	Number of SNPs
Exonic (stop gain)	182
Exonic (stop loss)	16
Exonic (synonymous)	62,247
Exonic (nonsynonymous)	32,117
Intronic	396,712
Splicing	207
Upstream	45,394
Downstream	36,563
Upstream/downstream	2,181
Intergenic	494,168
Unknown	6,991
ts	690,426
tv	386,352
ts/tv	1.787
Total	1,076,778

To generate the global perspective of the population stratification, we constructed the phylogenetic tree (**Figure S2a**) and performed the principal-component analyses (**Figure S2b**) and population structure analyses (**Figure S2c**). The results supported a division between LZ and HY populations, with some individuals overlap clustered. A possible reason is that the breeding parents from same ancestral populations were used in the two farming places. Furthermore, we also calculated the family structure, which could help reduce the false positives due to population stratification in GWAS analyses.

By calculating the pairwise LD between polymorphic sites, we found that LD decreased rapidly with increasing physical distance between markers from 0.026 (1 kb) to 0.08 (73 kb) (**Figure S2d**). The LD patterns also mirrored the genetic diversity of the LZ and HY populations.

### Genomic Regions and Loci Associated With Disease Resistance

**Figure 1:**
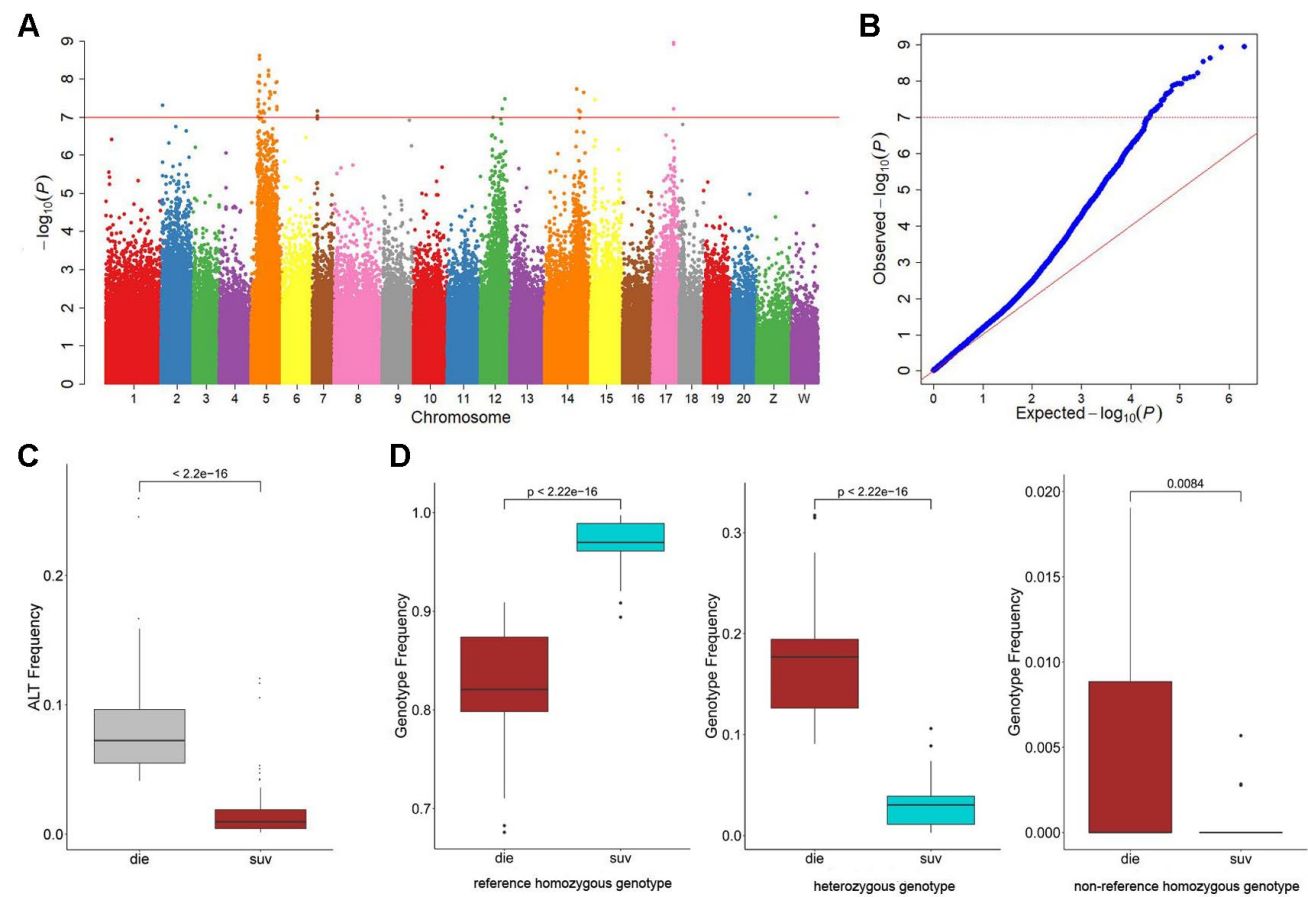
Genome-wide association study (GWAS) results of the resistance to *Vibrio harveyi* infection in *Cynoglossus semilaevis*. **(A)** Manhattan plot for binary phenotypes: survival and death. The red line indicates the Bonferroni-corrected significant threshold (−log_10_ *p* = 7). **(B)** Quantile-Quantile plot of the GWAS. **(C)** Boxplot for the mutational allele frequency of the GWAS identified single-nucleotide polymorphisms (SNPs). The statistical significance was calculated with the Wald test (*p* < 10^−35^). **(D)** Boxplots for the genotype frequencies of the GWAS identified SNPs.

To investigate the genetic variations underlying the disease resistance, we performed GWA mapping to identify the associated loci throughout the genome. Using the Bonferroni-corrected significance cutoffs (*P*-value = 10^−8^) yielded four associated loci (**Table 2**). Using *P*-Value of 10^−7^ as a cutoff threshold, a total of 33 SNPs were identified (**Table 2**, **Figures 1A, B**). We retained this permissive threshold of 10^−7^, corresponding to an α value of 0.1, in order to maximize the inclusion of suggestive candidates for further analysis. Occasionally we also consider SNPs meeting the less stringent threshold of 10^−6^, corresponding to an α value of 1. A tendency toward low P-values across the dataset (**Figure 1B**) precludes defining a precise significance threshold, so downstream results treat these SNPs as promising outliers rather than rigorously supported hits. Among the GWAS signals, 21 were located in Chr 5, and others were observed in Chr 2, 7, 12, 14, 15, and 17 (**Figure S3**), demonstrating a potentially clustered distribution of disease resistance associated signals among specific chromosomes. Additionally, we detected that these GWAS-obtained loci had significantly lower allele frequencies in the SUR group than in the DIE Group (*p* < 0.05) (**Figure 1C**), while no significant difference was observed on genomic level (*p* > 0.05) (**Figure S4A**). The most frequent genotype was reference homozygous allele (0/0) in both the SUR (Median Genotype Frequency = 0.982) and DIE group (median genotype frequency = 0.855) (**Figure 1D**), compared to the heterozygous allele and nonreference homozygous allele genotypes. The heterozygous genotype showed a significantly higher prevalence in the DIE Group, compared to the SUR group (*p* < 10^−16^), while the reference homozygous genotype frequency was significantly less in the DIE group than in the SUR Group (*p* < 10^−16^) (**Figure 1D**). Nonreference homozygotes were very rare, often unobserved, at all candidate GWAS SNPs (**Figure 1D**, **Table S4**), perhaps reflecting complex genetic structure (e.g. duplicated genes) or embryonic lethal selection against homozygotes, although we observed only mild and nonsignificant deviations from hardy–weinberg expectations. In contrast, genotype frequencies of all the loci throughout the genome were similar in the SUR and the DIE group (**Figure S4B**). These results indicate that the GWAS identified loci tends to fix the reference genotype in the SUR Group. Furthermore, to evaluate how the genetic drift impact the divergence, we simulated the allele frequency change of the alleles of the GWAS loci in DIE Groups over 25 generations. Across simulations, the median allele frequency varied slightly with time (**Figure S5**), and no significance change in allele frequencies was detected (t-test *p* > 0.05) between the first and other generations. These results indicated that very few genetic divergence were driven by genetic drift alone.

**Table 2:** The single-nucleotide polymorphisms (SNPs) significantly associated with the disease resistance.

SNP ID	Chr	Position	Ref	Alt	-log_10_ *P*	Peak effect	Gene ID	Gene annotation	% Var
CsSNP31*	chr17	13011075	C	A	8.95	Upstream	*fblx19*	F-box/LRR-repeat protein 19	2.68
CsSNP48	chr5	5066899	G	T	8.63	Intronic	*plekha7*	Pleckstrin homology domain-containing family A member 7	2.85
CsSNP49	chr5	5095129	A	T	8.53	Intergenic	*nucb2*	Nucleobindin-2	4.32
CsSNP76*	chr5	10827794	T	G	8.23	Intergenic	*rbl1*	Retinoblastoma-like protein 1	0.87
CsSNP42*	chr5	4427074	T	C	7.93	Intergenic	*usp10*	Ubiquitin carboxyl-terminal hydrolase 10	0.48
CsSNP102	chr5	16002435	G	C	7.92	exon,synonymous	*pde3b*	cGMP-inhibited 3′,5′-cyclic phosphodiesterase B	1.83
CsSNP97*	chr5	15676676	C	G	7.92	Intergenic	*st5*	Suppression of tumorigenicity 5 protein	1.47
CsSNP51	chr5	5256553	C	T	7.89	Intronic	*syt7*	Synaptotagmin-7	2.42
CsSNP66*	chr5	8456346	C	A	7.86	Intergenic	*cemip*	Cell migration-inducing and hyaluronan-binding protein	2.33
CsSNP47	chr5	5026823	G	T	7.69	Intergenic	N/A	N/A	0.76
CsSNP81	chr5	11704307	C	T	7.66	Intergenic	N/A	N/A	1.23
CsSNP96	chr5	14549123	C	T	7.65	Intergenic	N/A	N/A	2.08
CsSNP22	chr14	24494340	C	G	7.64	Intergenic	*psd3*	PH and SEC7 domain-containing protein 3	1.54
CsSNP83	chr5	12003828	G	T	7.60	Intergenic	*znt4*	Zinc transporter 4	5.32
CsSNP78*	chr5	11021059	C	T	7.51	Intergenic	N/A	N/A	1.64
CsSNP13*	chr12	15289909	C	G	7.47	Intronic	*sfr1*	Swi5-dependent recombination DNA repair protein 1 homolog	2.05
CsSNP26	chr15	2694305	T	C	7.47	Intergenic	*clnk*	Cytokine-dependent hematopoietic cell linker	3.61
CsSNP63	chr5	7976244	A	G	7.35	Upstream	*plekhg4b*	Pleckstrin homology domain-containing family G member 4B	2.53
CsSNP43*	chr5	4477882	C	T	7.35	Intergenic	N/A	N/A	2.79
CsSNP34	chr2	923324	G	T	7.32	Intronic	*ttll7*	Tubulin polyglutamylase TTLL7	1.76
CsSNP99	chr5	15846474	C	T	7.27	Intergenic	N/A	N/A	1.61
CsSNP11	chr12	13536810	G	A	7.23	Exon, synonymous	*b3galnt2*	UDP-GalNAc:beta-1,3-N-acetylgalactosaminyltransferase 2	3.25
CsSNP79*	chr5	11060924	C	T	7.23	Intergenic	N/A	N/A	1.05
CsSNP100	chr5	15869062	G	A	7.21	Intronic	*lmol*	Rhombotin-1	0.78
CsSNP19	chr14	21529897	C	T	7.17	Intergenic	*bmpr1b*	Bone morphogenetic protein receptor type-1B	1.58
CsSNP105	chr7	3016982	T	C	7.17	Intronic	*vsnl1*	Visinin-like protein 1	1.92
CsSNP61	chr5	7190601	C	T	7.16	Intergenic	*il16*	Pro-interleukin-16	1.83
CsSNP50	chr5	5160729	C	T	7.15	Intronic	*ext2*	Exostosin-2	1.12
CsSNP21	chr14	22391750	A	T	7.14	Intergenic	*tbx5*	T-box transcription factor TBX5	1.14
CsSNP64	chr5	8066080	C	T	7.11	Exon, synonymous	*adprhl1*	[Protein ADP-ribosylarginine] hydrolase-like protein 1	3.06
CsSNP55	chr5	5932644	C	T	7.11	Intergenic	*mapk8ip1*	C-Jun-amino-terminal kinase-interacting protein 1	1.49
CsSNP104	chr7	2965356	G	A	7.04	Intergenic	N/A	N/A	1.60
CsSNP5	chr12	7906314	G	A	7.00	Intronic	*nrxn1a*	Neurexin-1a-alpha	3.77

*SNPs located in candidate regions identified by Fst and nucleotide diversity filtration. %: Var, the proportion of the phenotypic variance explained by the SNP.

Using TASSEL, we calculated the proportion of phenotypic variance in disease resistance that could be explained by the associated SNPs. The proportion varied from 0.48% to 5.32%, suggesting a polygenic architecture affected by multiple loci with small effects (**Table 2**).

### Filtration of GWAS Results Using Fst and Nucleotide Diversity Analyses

**Figure 2:**
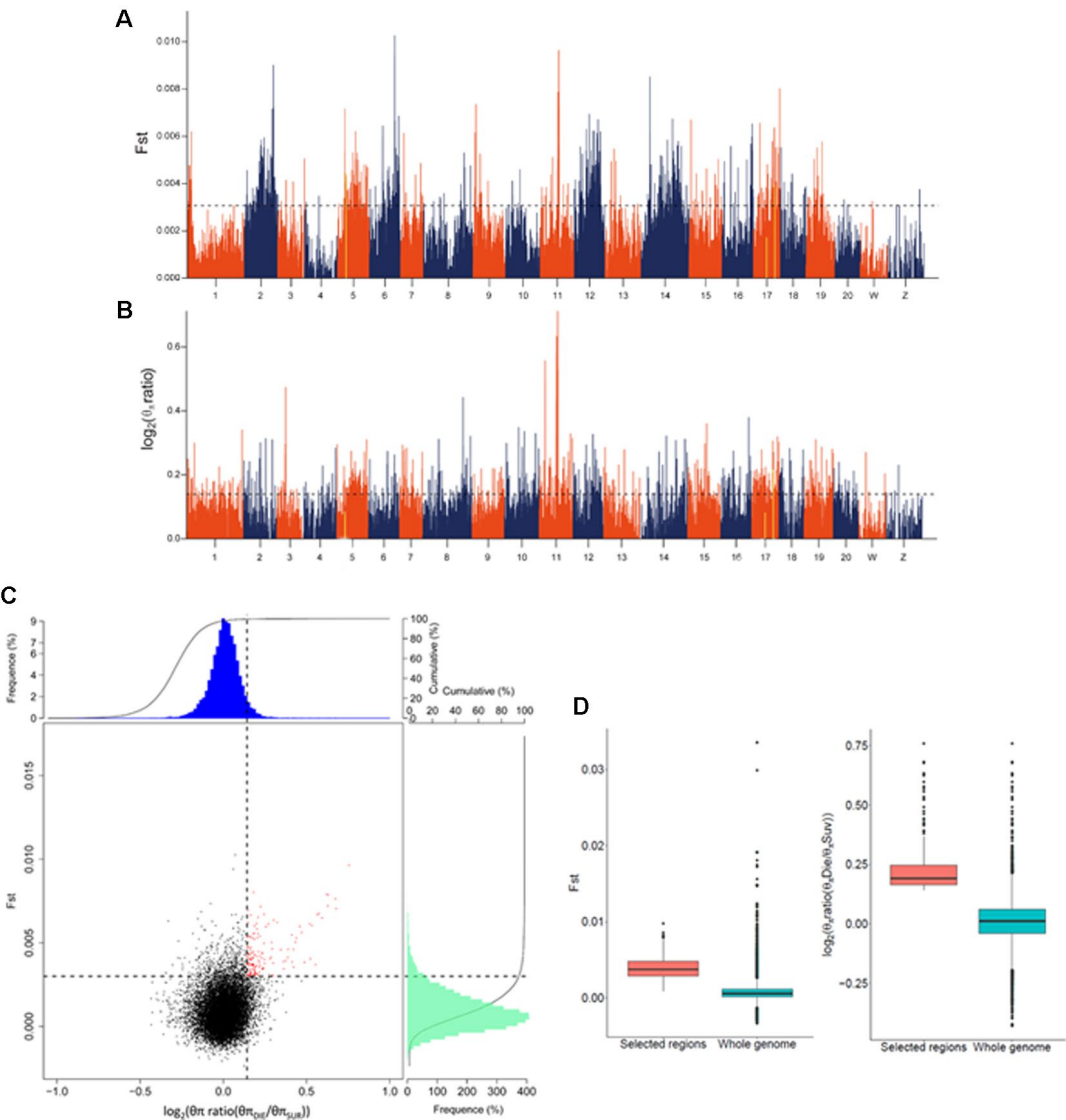
Genome-wide screening of genomic regions with Fst and nucleotide diversity estimation for disease resistance in *Cynoglossus semilaevis*. **(A)** Distributions of *Fst*, which were calculated in 40 kb windows with a 20 kb step. The dashed horizontal line corresponds to 5% top values. **(B)** Distributions of log_2_ [*θπ* ratio (*θπ*_DIE_/*θπ*_SUR_)]. The dashed horizontal line corresponds to 5% top values. **(C)** Distributions of log_2_ [*θπ* ratio (*θπ*_DIE_/*θπ*_SUR_)] and *Fst*. The red points (corresponding to the overlaps of top 5% log2 (θπ ratio) and Fst values) were identified as candidate regions. **(D)** The candidate regions exhibited a significantly higher *Fst* and log_2_ (*θπ* ratio), compared to the whole genomic background (*p* < 10^−16^).

To filter the GWAS identified loci, we examined two different indicators, the *Fst* and *θπ* ratio between the SUR and DIE group. Using a 40 kb window with 20 kb sliding steps across the genome, totally 120,979 windows were screened. We simultaneously used the top 5% highest values of *Fst* (where *Fst* = 0.003) (**Figure 2A**) and *θπ* ratio [where log_2_(*θπ* ratio _(_ *_θπ_*_DIE/_ *_θπ_*_SUR)_) = 0.143] (**Figure 2B**) as the thresholds. Consequently, 169 windows exhibited strong signals (**Figure 2C**). After merging the adjacent windows with a distance ≤200 kb, a total of 79 candidate regions with 16,940 SNPs, spanning 8.28 Mb genomic sequences were confirmed. These regions distributed in 15 chromosomes (**Figure S3**), containing 418 genes that belong to a diversity of functional categories (**Table S5**). The *Fst* and log_2_(*θπ* ratio) values of the candidate regions were significantly higher compared to those of the genomic background (*p* < 10^−16^) (**Figure 2D**).

### Candidate Disease Resistance Associated Genes in Candidate Regions

To further identify the loci, genes and regions accounting for the host resistance, we examined the overlaps between the GWAS signals and the candidate regions. Consequently, we detected a total of nine significantly associated SNPs (**Table 2**) residing in six candidate regions (**Table S6**), which provided additional supports for the genetic differentiation underlying the discrepancy of disease resistance in these genomic regions. A high proportion (77.8%, seven out of nine) of the SNPs were located in intergenic regions, suggesting regulatory implications of these loci. The other two SNPs were in intronic and upstream regions, respectively. The overlapped candidate regions were distributed in chromosome 5, 12, and 17 with lengths of 679.4, 29.9, and 40.0 kb, respectively, harboring 23 genes (**Table S7**).

**Figure 3:**
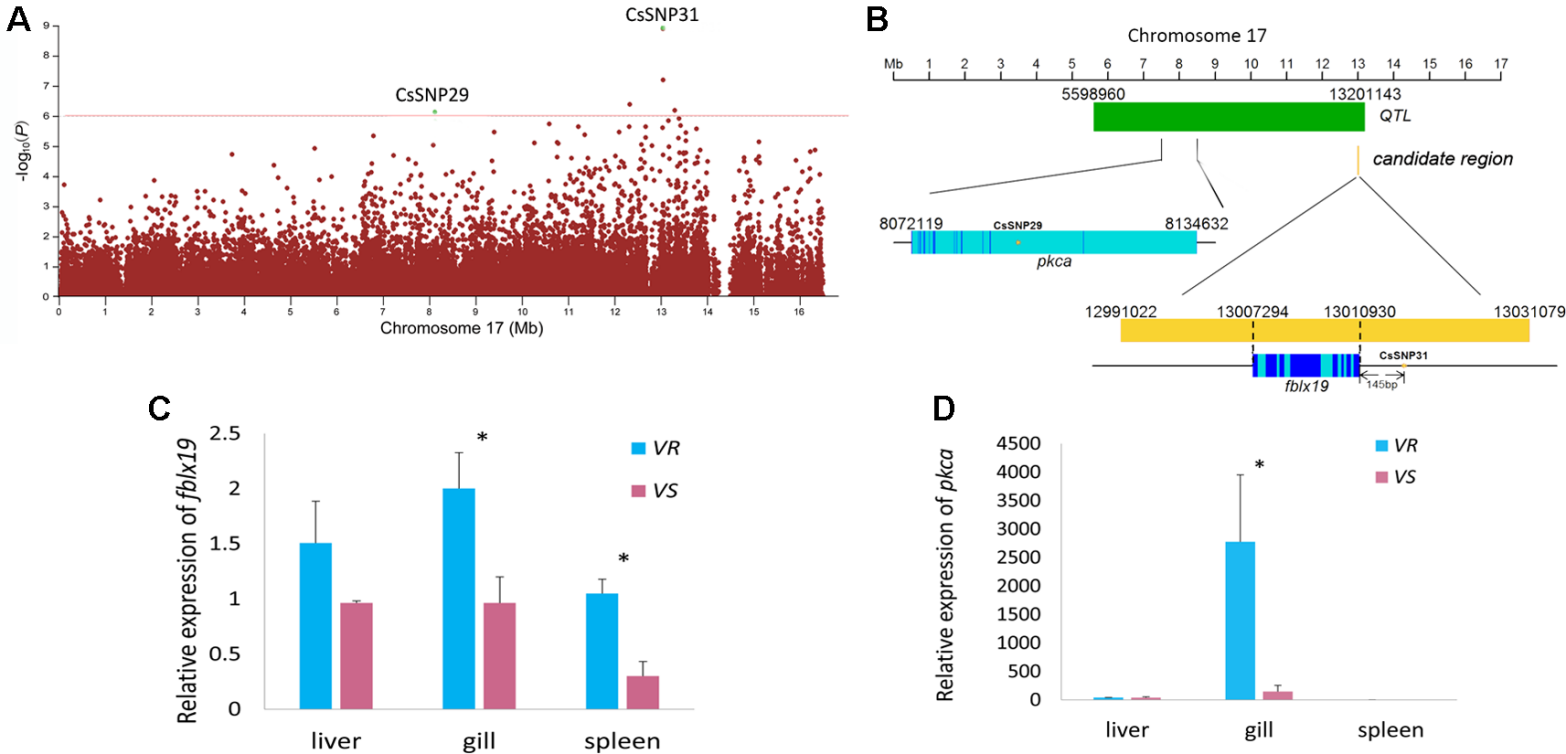
Genome-wide association study (GWAS) and Fst and nucleotide diversity filtration identified *fblx19* and *pkca* genes that are related to the disease resistance on Chr 17. **(A)** Regional Manhattan plot for Chr 17. The red line indicates the significance thresholds (−log_10_ *p* = 6). **(B)** The genomic positions of the GWAS-SNPs, *fblx19* and *pkca* gene. Dark blue, light blue, yellow and green bar represents exon, intron, candidate regions, and the disease resistance quantitative trait locus (QTL) previously identified (Dai et al., 2017), respectively. **(C**, **D)** Relative messenger RNA (mRNA) expression of *fblx19* and *pkca* gene in the *V. harveyi*-susceptible (VS) and *V. harveyi*-resistant (VR) families, detected by quantitative real-time PCR (qPCR) with *β*-actin gene as the internal control. The average values of three samples in each group were used to represent the expression level. Asterisks indicate significance difference (*p* < 0.05).

For the associated genes identified by GWAS and Fst and nucleotide diversity filtration, we used qPCR and BS-Seq to analyze their messenger RNA (mRNA) expressions and potential regulations by DNA methylation. The strongest GWAS signal CsSNP31 (−log_10_ *p* = 8.95) (**Figure 3A**) located in a candidate region in Chr 17 (Chr17: 12991022-13031079), occurring 145 bp upstream of F-box/LRR-repeat protein 19 gene (*fblx19*) (**Figure 3B**). Remarkably, this candidate region resides in a previously identified disease-resistant QTL (Chr 17: 5598960-13201143) (Dai et al., 2017) (**Figure 3B**). The proportion of phenotypic variance that could be explained by CsSNP31 was 2.68%. FBLX19 is a substrate-recognition component of the SCF (SKP1-CUL1-F-box protein)-type E3 ubiquitin ligase complex. It is reported to bind to the transmembrane interleukin 1 receptor and regulate its ubiquitination and degradation, thus plays a role in mammalian adaptive immune system (Zhao et al., 2012). Using qPCR analyses, we found that *fblx19* exhibited significantly higher expressions in the spleen and gill in the VR families than in the VS families (*p* < 0.05) (**Figure 3C**), indicating a potential function in the response to bacterial infection.

Another GWAS signal in Chr 17 (CsSNP29) (**Figure 3A**) with an association of −log_10_ *p* > 6 (**Table S8**), was located in intronic region of the protein kinase C alpha (*pkca*) gene, which also resides within the disease-resistant QTL (**Figure 3B**). PKC is a family of serine- and threonine-specific protein kinases that can phosphorylate a wide variety of protein targets and are involved in diverse cellular signaling pathways. The PKCA protein plays roles in many different cellular processes, such as cell adhesion, cell transformation, cell cycle checkpoint, and cell volume control (Nakashima, 2002; Haughian and Bradford, 2009). We compared the expression level of *pkca* gene in the VS and VR families, and observed that *pkca* was predominantly expressed in the gill and displayed approximately 18.9-fold higher expression in the VR families compared with in the VS families (**Figure 3D**). As an important regulator of multiple basic cellular processes, the significantly different expression of *pkca* indicated a distinctly different regulation of the cellular processes in the bacterial resistant and susceptible fish.

**Figure 4:**
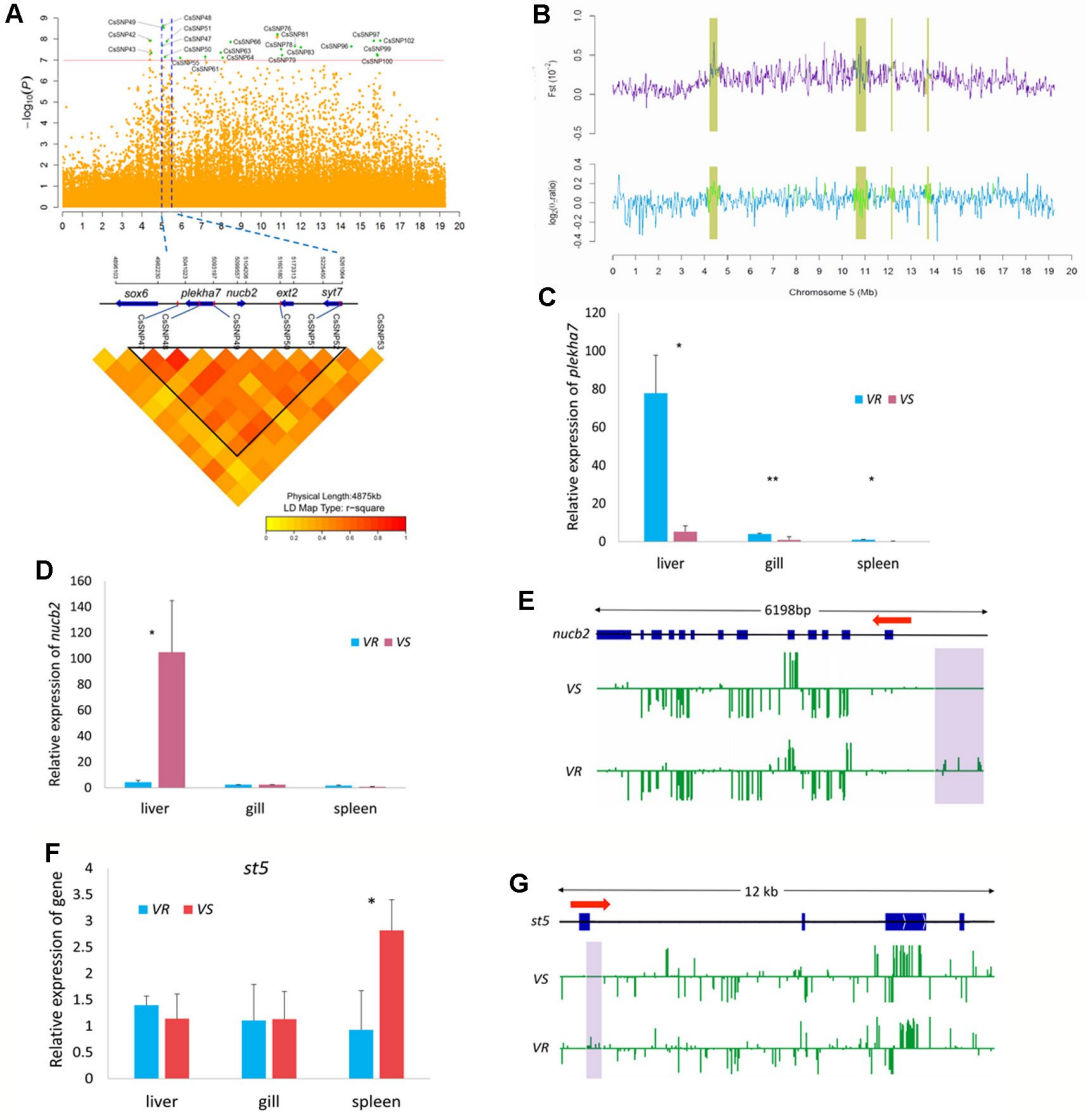
Genome-wide association study (GWAS) and Fst and nucleotide diversity filtration identified *plekha7* and *nucb2* gene were related to the disease resistance on Chr 5. **(A)** Manhattan plot (top) and local linkage disequilibrium (LD) heat map (bottom). Blue dotted lines indicate the candidate region between 5.01 and 5.26 Mb. CsSNP48 and CsSNP49 were located within or near *plekha7* and *nucb2* gene. **(B)** The candidate regions (yellow shades) identified by *Fs*t and nucleotide diversity (*θπ* ratio). **(C**, **D**, **F)** Relative messenger RNA (mRNA) expression of *plekha7* **(C)**, *nucb2* **(D)**, and *st5* **(E)** gene in the *V. harveyi*-susceptible (VS) and *V. harveyi*-resistant (VR) families, detected by quantitative real-time PCR (qPCR). The average values of three samples in each group were used to represent the expression level. Asterisks indicate significance difference (*p* < 0.05) and extremely difference (*p* < 0.01), respectively. **(E**, **G)** Comparison of the methylation levels of *nucb2* **(E)** and *st5* **(G)** gene in the VS and VR families. The genomic sequence of the gene body, the upstream and downstream area was analyzed. The arrow indicates the direction of transcription. The blue boxes indicate the exons. The methylation level of cytosines is shown with green vertical lines and the violet shades indicate the significantly different methylation regions.

In Chr 5, we identified 22 significantly associated SNPs (66.7% out of all association peaks) (**Figure 4A**) and 15 candidate regions (occupying 29.2% out of total length of all candidate regions) (**Figure 4B**). We focused on the loci mapped from 5.01 to 5.26 Mb, with five associated SNPs (CsSNP47–CsSNP51) and four genes (**Figure 4A**). A strong association signal CsSNP48 (−log_10_ *p* = 8.63) occurred in an intron of the gene encoding Pleckstrin homology domain-containing family A member 7 (PLEKHA7). This SNP could explain 2.85% of the phenotypic variance. PLEKHA7 is a cytoplasmic member of the adheres junction proteins, which may induce apoptosis through the lysosomal–mitochondrial pathway. Interestingly, it plays an important role in controlling susceptibility to *Staphylococcus aureus* α-toxin and was identified as a potential nonessential host target to reduce *S. aureus* virulence during epithelial infections (Popov et al., 2015). Quantitative PCR analyses showed that the expressions of *plekha7* was significantly higher in the immune organs of the VR families than in the VS families (**Figure 4C**). Another highly associated SNP, CsSNP49 (−log_10_ *p* = 8.53), was mapped to the intergenic region of *plekha7* and nucleobindin-2 (*nucb2*) gene. The proportion of phenotypic variance explained by this SNP was 4.32%. *Nucb2* encodes a calcium-binding protein that may have a role in calcium homeostasis and plays an important role in hypothalamic pathways regulating food intake and energy homeostasis (Foo et al., 2008). Increased plasma NUCB2 concentrations are closely associated with inflammation, trauma severity, and clinical outcomes, indicating that NUCB2 might be involved in inflammation and is a potential biomarker for diseases in human (Ramesh et al., 2017). qPCR analyses revealed that the transcript level of *nucb2* was significantly higher in the liver of the VS families than in the VR families (*p* < 0.05) (**Figure 4D**). Furthermore, we observed that the promoter region of the *nucb2* gene was upmethylated in the VR families compared with the VS families (*p* < 0.05) (**Figure 4E**). These results suggested a role of *nucb2* in bacterial susceptibility, which may be regulated by DNA methylation.

Notably, up to 15 GWAS SNPs were located in intergenic regions, implying that multiple regulations may be involved in the disease resistance (**Table 2**). Furthermore, several SNPs and related genes were located in or near the candidate regions, providing additional evidence for genomic differentiation associated with response to bacterial infection. For example, the suppression of tumorigenicity 5 (*st5)* gene that encodes a guanine nucleotide exchange factor, was within 5 kb distance to a strong association signal CsSNP97 (−log_10_ *p* = 7.919) (**Figure 4A**). *St5* is involved in regulating the MAPK1/ERK2 signaling transduction pathway and cell morphology (Majidi et al., 1998). We observed that s*t5* displayed a higher expression in the spleen of the VS families than in the VR families (**Figure 4F**), and its first exonic and intronic regions were upmethylated in the VR families (**Figure 4G**).

Even though not highly significant, we observed a GWAS signal (CsSNP10, −log_10_ *p* > 6) in an intron of fibroblast growth factor receptor 2 (*fgfr2*) gene (**Figure S6A** and **Table S7**), explaining 3.08% of the phenotypic variation and residing in a candidate region (**Figure S6B**). *Fgfr2* encodes a tyrosine-protein kinase that acts as cell-surface receptor for fibroblast growth factors. Ligand binding of this protein leads to activation of several signaling cascades and it plays an essential role in the regulation of cell proliferation, differentiation, migration, and apoptosis (Liu et al., 2017). The *fgfr2* gene displayed significantly higher expressions in the liver and the gill of the VR families than in the VS families (**Figure S6C**). Corresponding to the expression difference, the promoter region of *fgfr2* in the VS families was upmethylated in comparison with in the VR families, indicating the DNA methylation may play a role in the expression regulation of this gene (**Figure S6D**).

## Discussion

Phenotypic variations in desirable traits have been observed in selective breeding programs. The dramatic changes in disease resistance to *V. harveyi* with three to four generations of selection in *C. semilaevis* indicated that the artificial selection might act on the evolution of disease resistance. We explored the genetic patterns and genomic structure of the disease resistance using genome-wide genetic analyses, holding application implications in genetic engineering and broodstock cultivation.

To date, most of the reported genetic and association studies were performed using restriction-site associated DNA sequencing (RAD-Seq) or SNP chip (reviewed in Robledo et al., 2018), with limited number of SNP genotypes produced. With these methods, the detected QTLs could span relative large genomic regions, making it difficult to accurately identify the causative loci and genes. Our GWAS and candidate region identification were performed based on whole genome resequencing data, which predicted large number of SNPs throughout the genome, and allowed fine-mapping of the associated loci and genes. Although deep sequencing of single individuals is generally required to generate accurate calls, it was reported that with hundreds of individuals, accurate genotype calls can be generated for a low sequence depth (Le and Durbin, 2011; Li et al., 2011).

Association studies have been applied to detect a number of causative and associated loci and genes for diseases resistance in fish. For instance, many candidate genes involved in PI3K pathway are reported to be significantly associated with columnaris resistance in catfish (Geng et al., 2015). Multiple loci and genes were reported to play an important role in the immune response against *Piscirickettsia Salmonis* (Correa et al., 2015) and sea lice (Robledo et al., 2019) in Atlantic salmon, pasteurellosis in gilthead sea bream (Palaiokostas et al., 2016), and *V. anguillarum* in Japanese flounder (Shao et al, 2015). In addition, major QTLs were identified for bacterial cold water disease (Vallejo et al, 2017) and resistance to myxosporean parasite *Myxobolus cerebralis* in rainbow trout, pancreas disease (Gonen et al., 2015), and amoebic gill disease in Atlantic salmon (Robledo et al., 2018). Our GWAS result supported that the disease resistance trait is polygenic in nature. We detected a tendency towards reference alleles in the resistant group, probably because the reference genome assembly we used for the SNP calling was generated from a resistant individual. In addition, the significantly different allele and genotype frequencies between the surviving and the dead fish make the GWAS-polymorphisms a very informative and valuable marker system to evaluate whether the inheritance of these variants could result in or have impact on the resistance to pathogens. The identified resistant alleles can be of significant value to the selective breeding and could be used for protecting fish against disease.

We also found low but significant genetic divergence, which was also reported in natural populations of Atlantic salmon (Aykanat et al., 2015). These results indicate that low genetic differentiation could indeed be meaningful for biological evolution and mechanisms. Genes in the identified candidate regions showed diverse functional categories and more studies are needed to clarify their roles in the disease resistance.

Combined analyses of GWAS and Fst and nucleotide diversity filtration narrowed and highlighted the meaningful genomic regions, and supported the functional significance of the loci and genes within these regions. A considerable number of the overlapping GWAS loci and candidate regions were located in noncoding genomic region, indicating that regulatory elements may play important roles in the disease resistance of *C. semilaevis*. The colocalization of the GWAS signal, candidate regions and QTL further verified the power of the combined analyses. Moreover, a high proportion of the associations and candidate regions were present in Chr 5, implying the importance of this chromosome in the disease resistance.

The candidate genes we identified could be potential targets for genetic improvement aiming at enhancing the disease resistance. To our knowledge, most of the candidate genes, such as *fblx19*, *plekha7*, and *nucb2*, were reported to be related to disease resistance in fish for the first time. Differential expression and methylation were observed in some genes between resistant and susceptible families. Nevertheless, some candidate genes did not exhibit any significant difference in the mRNA levels. A possible reason is that the genetic variations may have other consequences that could not be reflected by the mRNA expression. Also, for quantitative traits like disease resistance, the effect contributed by a single gene or a locus may be very low. Alternatively, other nearby or remote genes may be connected to the loci and contribute to the disease resistance. More functional analyses, such as genome editing, will help validate the role of these genes and loci in the disease resistance.

Our findings provide useful information for the breeding programs and stock management of farmed fish. The comprehensive analyses starting at a genetic level allows a better understanding of the evolution and genetics of disease resistance in fish. The identified genes and loci are useful targets for genetic improvement and genomic breeding programs, which is of a high value for application in the breeding activity in a wide range of aquaculture populations. In the future, deep genotyping of larger samples will be necessary to identify low frequency or rare mutations and to link GWAS results with multiple outcomes. Another extension to our work would be elucidating the function of the identified alleles and genes, and the regulatory mechanisms underlying the disease resistance in fish.

## Data Availability Statement

Raw reads from Illumina sequencing are deposited in the NCBI Sequence Read Archive (SRA) database with Bioproject accession PRJNA542202.

## Ethics Statement

This study was carried out in accordance with the recommendations of the Care and Use of Laboratory Animals of the Chinese Academy of Fishery Sciences. The protocol was approved by the Animal Care and Use Committee of the Chinese Academy of Fishery Sciences.

## Author Contributions

SC and QZ conceived the study and designed the analytical strategy. YLi, FL, YLiu, LW, XZ and SL performed animal work and prepared biological samples. QZ, ZS, and GL analyzed the data. SW, TG, and MW performed the qPCR experiments. QZ, SC and GL wrote the manuscript. All authors reviewed the manuscript.

## Funding

This work was supported by National Natural Science Foundation of China (grant number 31530078, 31730099); National Key R&D Program of China (grant number 2018YFD0900301); AoShan Talents Cultivation Program Supported by Qingdao National Laboratory for Marine Science and Technology (grant number 2017ASTCP-OS15) and Taishan Scholar Climbing Project of Shandong Province of China.

## Conflict of Interest

Authors ZS, XZ, and GL was employed by Novogene Bioinformatics Technology Co., Ltd.

The remaining authors declare that the research was conducted in the absence of any commercial or financial relationships that could be construed as a potential conflict of interest.

## Footnotes

Retrieved March 2019
