# Supplementary material for: Genome-Wide Association Mapping and Gene Expression Analyses Reveal Genetic Mechanisms of Disease Resistance Variations in Cynoglossus semilaevis
Source: Front Genet. 2019 Nov 20;10:1167. doi: 10.3389/fgene.2019.01167 (PMC6880758; doi:10.3389/fgene.2019.01167)
Supplement: Supplementary file 1 [file DataSheet_1.docx]

**Genome-wide association mapping and gene expression analyses reveal genetic mechanisms of disease resistance variations in *Cynoglossus semilaevis***

Qian Zhou^1,2,3,$^, Zhencheng Su^4,$^, Yangzhen Li^1^, Yang Liu^1^, Lei Wang^1^, Sheng Lu^1^, Shuanyan Wang^1^, Tian Gan^1^, Feng Liu^1^, Xun Zhou^4^, Min Wei^1^, Guangjian Liu^4*^, Songlin Chen^1,2,3*^

**Supplementary Materials**

**
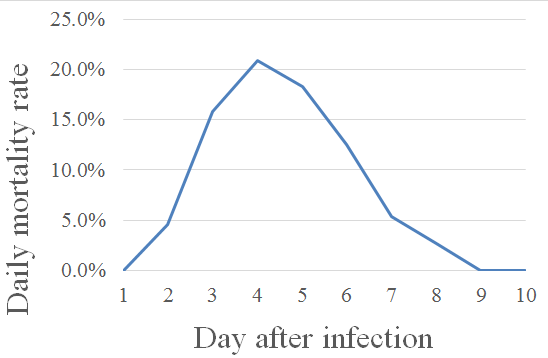
**

**Figure S1** Daily mortality rates after *V. harveyi* infection.

**
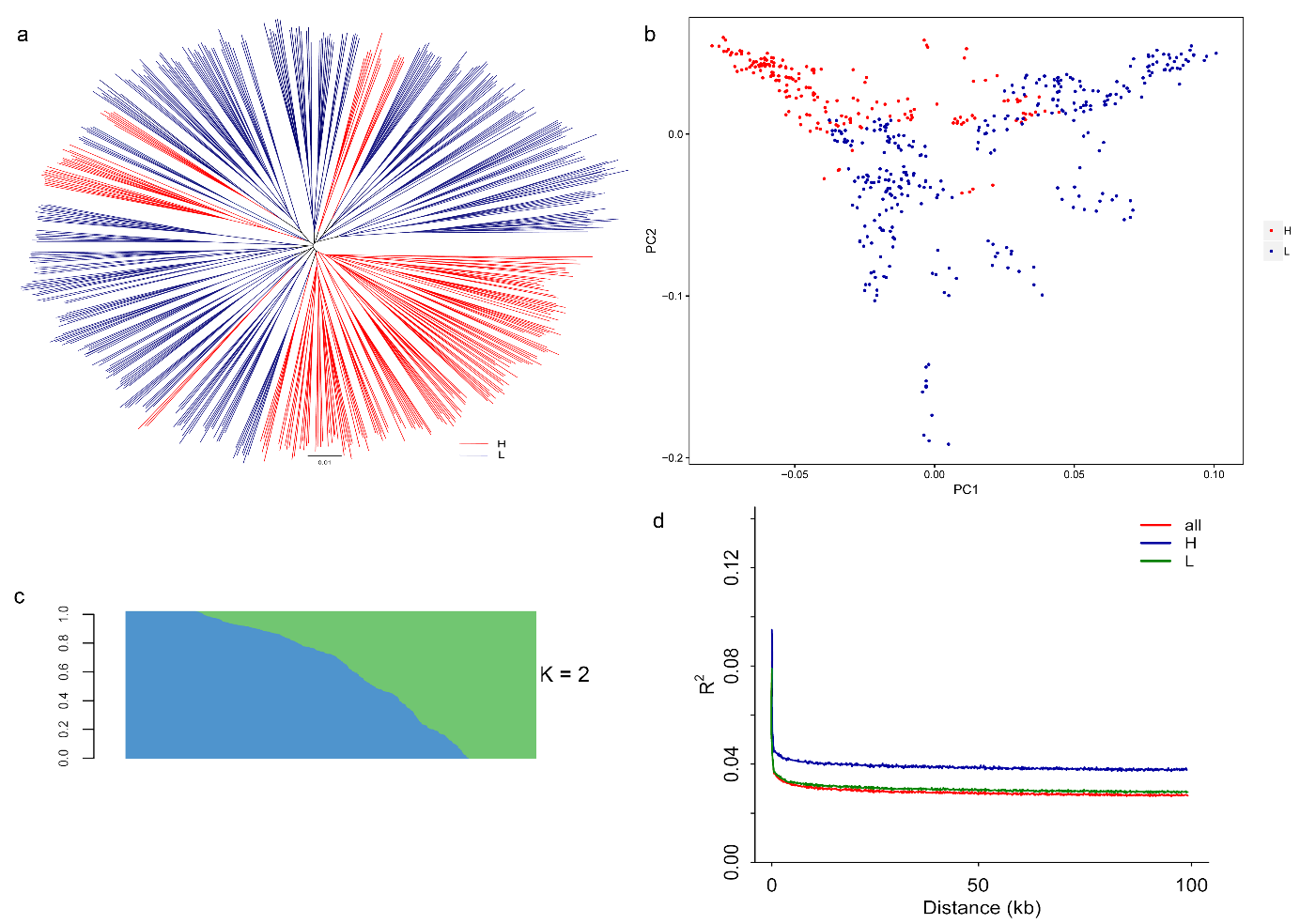
**

**Figure S2** The phylogenetic relationship and LD decay. (a) A neighbor-joining tree constructed using the SNP data. H: Haiyang population, L: Laizhou population. (b) PCA of the 505 individuals. The plot shows the first two principal components. (c) Population structure plots with K = 2. Individuals are represented as rows partitioned into segments corresponding to the inferred membership as indicated by the colors. (d) LD decay determined by squared correlations of allele frequencies (r^2^) against distance between polymorphic sites in SUR (blue) and DIE (red) groups.


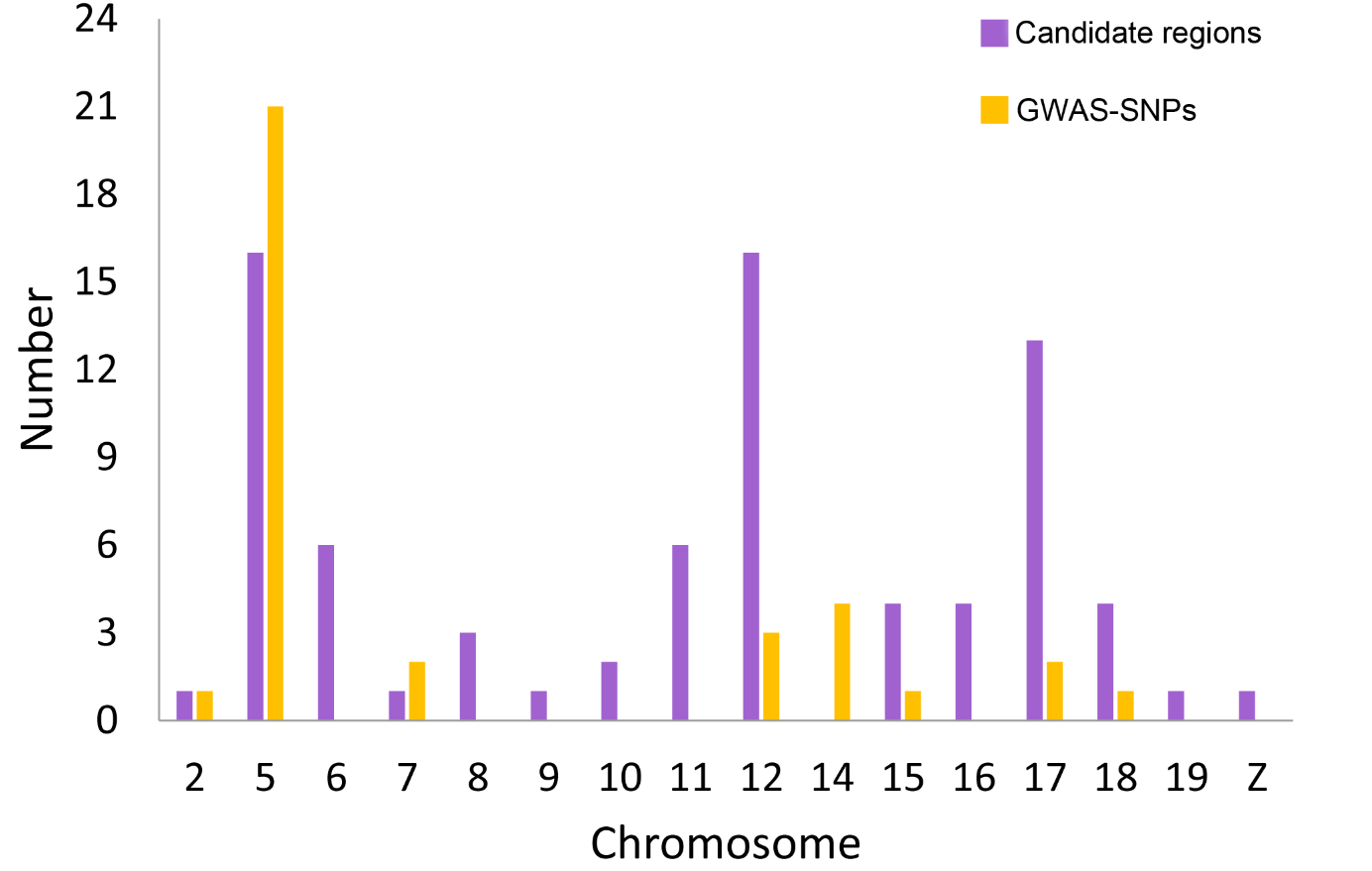


**Figure S3** Distribution of significantly associated SNPs and candidate regions identified by Fst and nucleotide diversity filtration to disease resistance in the genome of *C. semilaevis*.

**
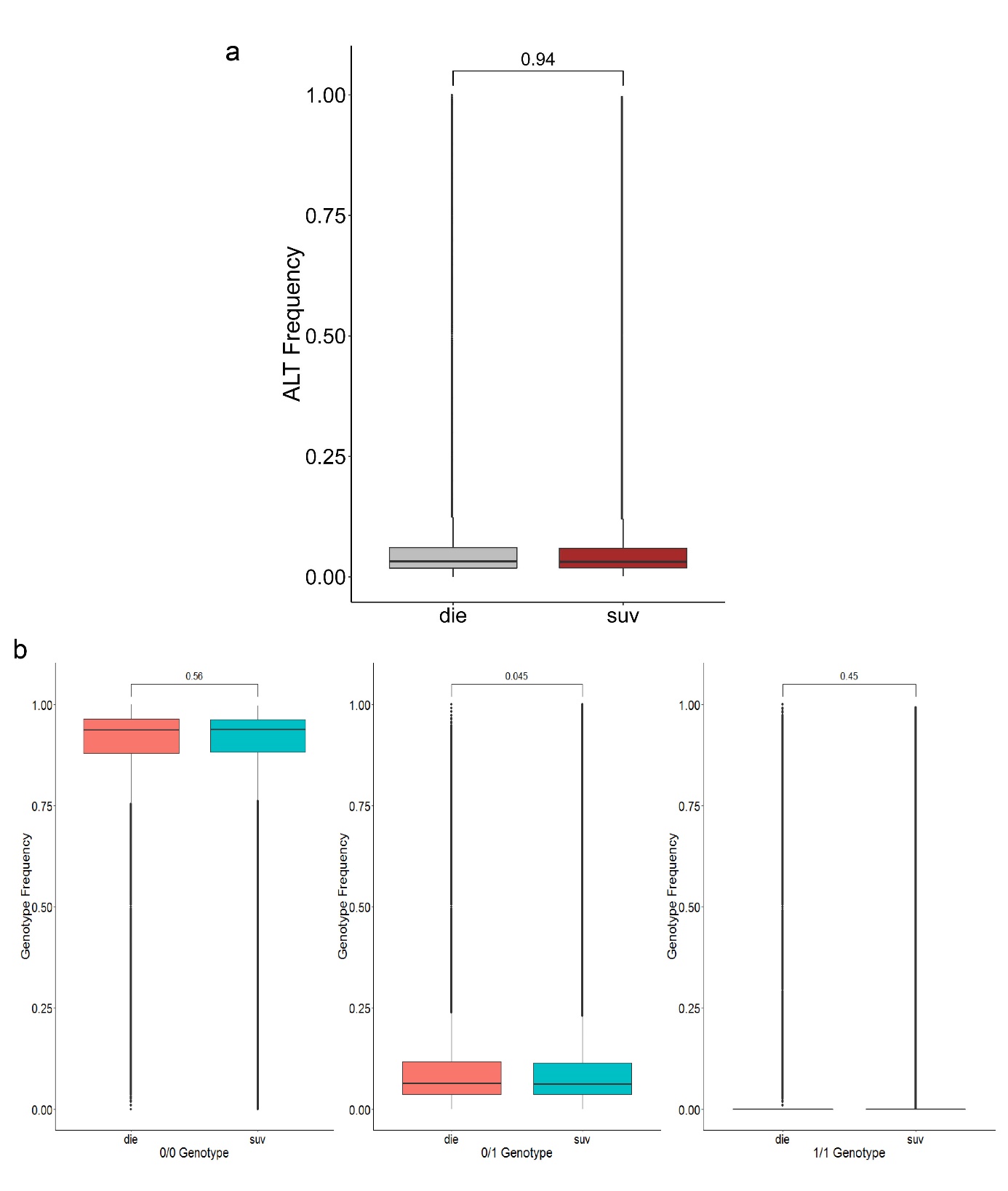
Figure S4** Boxplots for the (a) allele frequency and (b) genotype frequencies of genome-wide SNPs in SUR and DIE group, respectively. No statistical significance was observed with the Wald test.


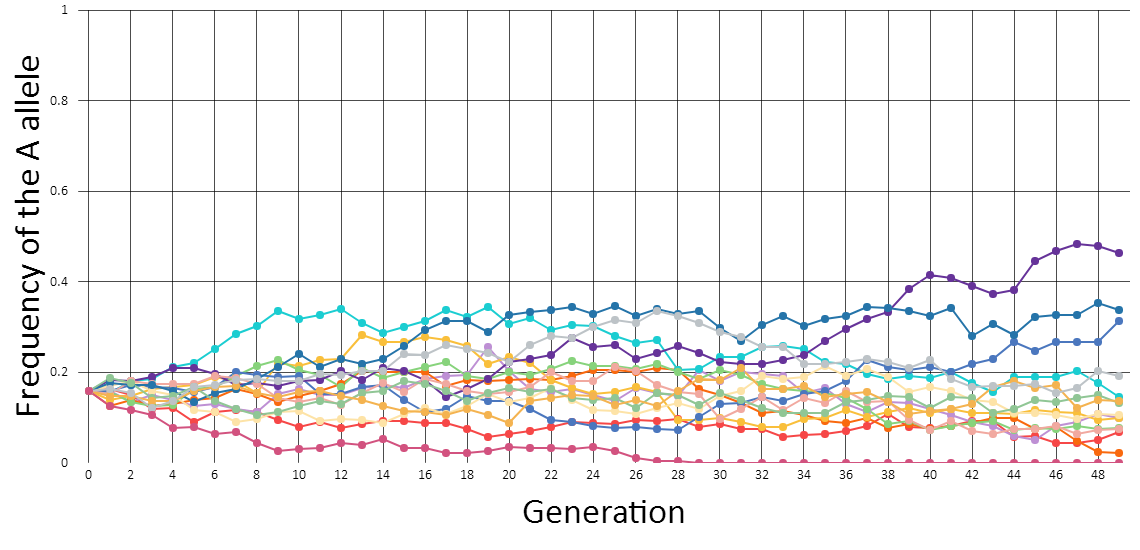


**Figure S5** Genetic drift simulation for the significantly associated allele with 2.3% allele frequency (carrier frequency: 4.6%) over 50 generations. Plot shows frequency after 50 generations for 10 simulations. The allele is lost in approximately 62% of the simulations, while in 25% of the simulations the frequency after 25 generations is equal or greater than the start frequency.


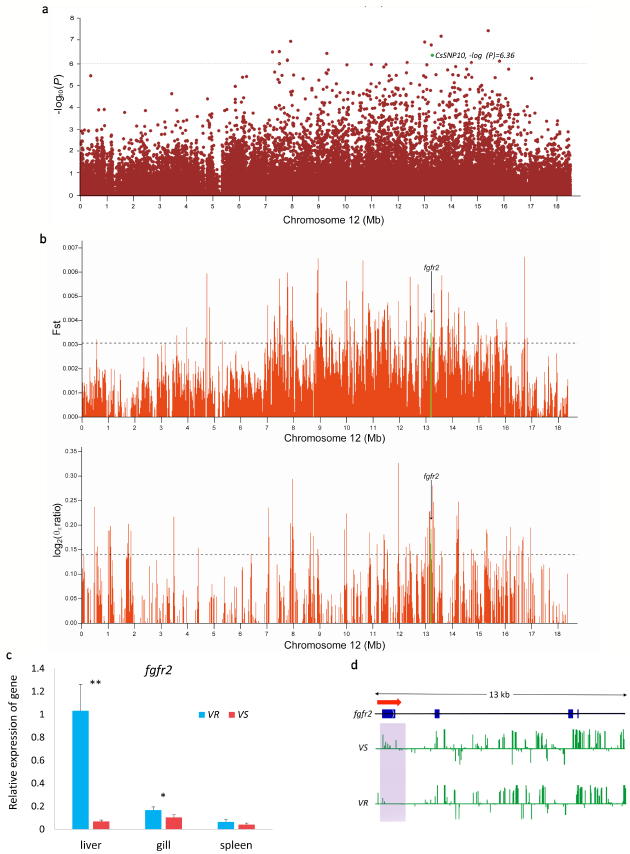


**Figure S6** GWAS and filtration using Fst and nucleotide diversity identified that *fgfr2* gene was related to the disease resistance on Chr 17. (a) Manhattan plot for Chr 12. The dashed line indicates the significance thresholds (-log_10_*P* = 6). (b) Distributions of *Fst* and log_2_ (*θπ* ratio (*θπ*_DIE_/*θπ*_SUR_)). The dashed lines correspond to top 5% distribution. (c) Relative mRNA expression of *fgfr2* gene in the VS and VR families, detected by qPCR with *β*-actin gene as the internal control. The average values of three samples in each group were used to represent the expression level. Asterisks indicate significance difference (*p* < 0.05). (d) Methylation of *fgfr2* gene. The genomic sequence of the gene body, upstream and downstream area was analyzed. The arrow indicates the direction of transcription. The blue boxes indicate the exons. The methylation level of cytosines is shown with green vertical lines and the violet shade indicates the significantly different methylation regions.

**Table S1. Primer sequences of qPCR used in this study.**

| **Primer** | **Sequence (5′-3′)** |
| --- | --- |
| fblx19-F | CTTCAGGTGGAGGTAGCAGC |
| fblx19-R | CTGGGATGGATGATCTCGTT |
| fgfr2-F | TGAAGACGCATCGAACAAATAC |
| fgfr2-R | TGAGGTCCAATACGGAGCAC |
| nucb2-F | TCCTGCTGATGGTCCACTTC |
| nucb2-R | GCTCACCTCTTGCCTCTTTA |
| pkca-F | CCAGATCCAAAGAATGAAACCA |
| pkca-R | GCTCAGAAACGCCAAACGA |
| plekha7-F | AAGCACCACTTGGCTCCATC |
| plekha7-R | TGACTTGGACATGCGGAATT |
| st5-F | GTTCTCTCAGCCAGTCAGGG |
| st5-R | CCTGTTTCTCCTTGGTTTGA |
| β-actin-F | TTCCAGCCTTCCTTCCTT |
| β-actin-R | TACCTCCAGACAGCACAG |

**Table S2. The genotype of GWAS identified SNPs in the 12 individuals used in qPCR analysis.**

| **#CHROM** | **POS** | **REF** | | | **ALT** | | **VR1-1** | **VR1-2** | **VR1-3** | **VR2-1** | **VR2-2** | **VR2-3** | **RS1-1** | **RS1-2** | **RS1-3** | **RS2-1** | **RS2-2** | **RS2-3** |
| --- | --- | --- | --- | --- | --- | --- | --- | --- | --- | --- | --- | --- | --- | --- | --- | --- | --- | --- |
| chr5 | 15676676 | | C | G | | C | | C | C | C | C | C | C/G | C/G | C/G | C | C/G | C |
| chr5 | 5066899 | G | | | T | | G | G | G | G | G | G | G/T | G/T | G/T | G | G | G/T |
| chr5 | 5095129 | A | | | T | | A | A | A | A | A | A | A/T | A/T | A/T | A/T | A | A |
| chr17 | 13011075 | C | | | A | | C | C | C | C | C | C | C | C | C | C/A | C/A | C |
| chr17 | 8095454 | C | | | T | | C | C | C | C | C | C | T | T | T | T | T | T |

**Table S3. Statistics of the genome resequencing data of 505 individuals of *C.semilaevis*.**

| **Sample** | **Raw Base (bp)** | **Clean Base (bp)** | **Clean reads** | **Mapped reads** | **Mapping rate (%)** | **Sequencing depth** | **Coverage** |
| --- | --- | --- | --- | --- | --- | --- | --- |
| L19-1 | 1207596600 | 1184196900 | 7894646 | 5747575 | 72.8 | 2.41 | 66.31 |
| H63-3 | 1029630600 | 1007691600 | 6717944 | 5145312 | 76.59 | 2.27 | 63.49 |
| L36-10 | 1304422200 | 1283596500 | 8557310 | 6691511 | 78.2 | 2.66 | 70.84 |
| L40-8 | 1308203400 | 1279611600 | 8530744 | 6934583 | 81.29 | 2.74 | 71.24 |
| L75-9 | 897782400 | 890200800 | 5934672 | 5014655 | 84.5 | 2.2 | 65.18 |
| L69-1 | 1493863200 | 1476511200 | 9843408 | 8364482 | 84.98 | 3.03 | 77.51 |
| L6-3 | 1259023200 | 1228092900 | 8187286 | 6968713 | 85.12 | 2.7 | 71.79 |
| L24-7 | 1232765700 | 1206623100 | 8044154 | 6849166 | 85.14 | 2.71 | 70.29 |
| H62-7 | 1072192500 | 1051712100 | 7011414 | 5997224 | 85.54 | 2.41 | 68.44 |
| L3-4 | 1254317100 | 1225223400 | 8168156 | 7002027 | 85.72 | 2.71 | 71.94 |
| L36-6 | 1474676100 | 1462727100 | 9751514 | 8408278 | 86.23 | 3.03 | 78.08 |
| H49-2 | 1291756200 | 1265913300 | 8439422 | 7294291 | 86.43 | 2.75 | 73.01 |
| H50-25 | 1296527100 | 1266712200 | 8444748 | 7307587 | 86.53 | 2.74 | 72.57 |
| L21-1 | 1416535500 | 1391236500 | 9274910 | 8058724 | 86.89 | 2.9 | 76.89 |
| L69-9 | 1080795900 | 1071764400 | 7145096 | 6217262 | 87.01 | 2.5 | 71.3 |
| H13-21 | 1105488300 | 1079415300 | 7196102 | 6263055 | 87.03 | 2.51 | 69.73 |
| L2-8 | 1161396300 | 1141095300 | 7607302 | 6638163 | 87.26 | 2.59 | 71.42 |
| H26-21 | 1187361300 | 1043462400 | 6956416 | 6087314 | 87.51 | 2.56 | 65.74 |
| L69-8 | 1401114000 | 1390798800 | 9271992 | 8123788 | 87.62 | 2.91 | 78.98 |
| L110-1 | 983955900 | 970165200 | 6467768 | 5684273 | 87.89 | 2.38 | 67.42 |
| H27-23 | 1000070400 | 965227500 | 6434850 | 5663308 | 88.01 | 2.46 | 64.48 |
| H6-15 | 1219396500 | 1180379100 | 7869194 | 6928023 | 88.04 | 2.56 | 69.51 |
| H36-25 | 1041861000 | 1015941000 | 6772940 | 5971518 | 88.17 | 2.45 | 67.88 |
| L36-4 | 1250558400 | 1230039600 | 8200264 | 7234301 | 88.22 | 2.85 | 71.94 |
| H26-13 | 1181805300 | 1152534600 | 7683564 | 6788511 | 88.35 | 2.62 | 71.34 |
| L2-10 | 1497535500 | 1463934900 | 9759566 | 8651805 | 88.65 | 3.1 | 77.74 |
| H44-25 | 1110645000 | 1081751700 | 7211678 | 6398734 | 88.73 | 2.65 | 66.56 |
| H26-25 | 1030830300 | 1007804400 | 6718696 | 5964630 | 88.78 | 2.53 | 66.17 |
| L68-8 | 1391907300 | 1356281700 | 9041878 | 8034244 | 88.86 | 3.04 | 74.97 |
| L37-5 | 1172037600 | 1163738700 | 7758258 | 6902288 | 88.97 | 2.74 | 71.74 |
| H26-11 | 1112449800 | 1081932300 | 7212882 | 6418878 | 88.99 | 2.58 | 70.5 |
| H8-21 | 1118856300 | 1089514200 | 7263428 | 6466109 | 89.02 | 2.56 | 69.31 |
| L53-1 | 1143426000 | 1117581300 | 7450542 | 6638912 | 89.11 | 2.6 | 72.31 |
| H24-45 | 1003891500 | 974133000 | 6494220 | 5797196 | 89.27 | 2.36 | 64.22 |
| L19-21 | 1328956800 | 1295217900 | 8634786 | 7711495 | 89.31 | 2.86 | 74.03 |
| L59-5 | 1468402800 | 1434078900 | 9560526 | 8544727 | 89.38 | 3.14 | 76.56 |
| H47-21 | 1079178900 | 1051226700 | 7008178 | 6283185 | 89.66 | 2.51 | 69.32 |
| L74-3 | 1302510600 | 1279626600 | 8530844 | 7664772 | 89.85 | 2.92 | 74.42 |
| L52-8 | 1088820600 | 1077714900 | 7184766 | 6467749 | 90.02 | 2.55 | 72.29 |
| L3-21 | 1034770500 | 1014197400 | 6761316 | 6089188 | 90.06 | 2.49 | 68.4 |
| L36-8 | 1447467900 | 1431659400 | 9544396 | 8632234 | 90.44 | 3.08 | 77.33 |
| H73-2 | 1430554500 | 1406949300 | 9379662 | 8483712 | 90.45 | 3.09 | 76.14 |
| L70-9 | 932504100 | 924393000 | 6162620 | 5574124 | 90.45 | 2.33 | 67.99 |
| H35-1 | 1338032700 | 1309055700 | 8727038 | 7894812 | 90.46 | 2.92 | 75.96 |
| L35-7 | 1338409800 | 1325995200 | 8839968 | 8055558 | 91.13 | 2.94 | 76.81 |
| L3-3 | 1201608000 | 1161632100 | 7744214 | 7058020 | 91.14 | 2.69 | 71.88 |
| L109-1 | 1350603600 | 1327426800 | 8849512 | 8067059 | 91.16 | 2.9 | 76.36 |
| L3-10 | 1069565100 | 1055737800 | 7038252 | 6421445 | 91.24 | 2.51 | 72.19 |
| L74-9 | 1066483200 | 1021786200 | 6811908 | 6234378 | 91.52 | 2.59 | 67.44 |
| L56-11 | 1359832200 | 1326415800 | 8842772 | 8099443 | 91.59 | 2.97 | 73.4 |
| L75-6 | 1155390000 | 1134590100 | 7563934 | 6932073 | 91.65 | 2.62 | 72.39 |
| L52-4 | 1042931400 | 1030558200 | 6870388 | 6302211 | 91.73 | 2.55 | 70.84 |
| L46-10 | 1412942400 | 1395887100 | 9305914 | 8551410 | 91.89 | 3.12 | 77.14 |
| L35-6 | 1185989400 | 1171237800 | 7808252 | 7182614 | 91.99 | 2.78 | 73.14 |
| L28-3 | 1064312700 | 1043130900 | 6954206 | 6422738 | 92.36 | 2.62 | 69.72 |
| H34-1 | 1133339100 | 1108317000 | 7388780 | 6825527 | 92.38 | 2.65 | 71.45 |
| L18-1 | 1285403400 | 1263666000 | 8424440 | 7783109 | 92.39 | 2.78 | 72 |
| L74-5 | 1149257100 | 1137531600 | 7583544 | 7026935 | 92.66 | 2.67 | 73.36 |
| L36-3 | 1179080700 | 1158797700 | 7725318 | 7163254 | 92.72 | 2.8 | 72.68 |
| L13-21 | 1078855200 | 1055337000 | 7035580 | 6525940 | 92.76 | 2.47 | 68.32 |
| L3-5 | 1175911500 | 1162358700 | 7749058 | 7193391 | 92.83 | 2.73 | 71.4 |
| L69-6 | 1583298000 | 1568591400 | 10457276 | 9708130 | 92.84 | 3.31 | 81.56 |
| L3-6 | 1627265700 | 1594700400 | 10631336 | 9871467 | 92.85 | 3.48 | 79.56 |
| L28-6 | 1106864400 | 1090108500 | 7267390 | 6755996 | 92.96 | 2.67 | 72.21 |
| L75-10 | 1046771700 | 1007840100 | 6718934 | 6252217 | 93.05 | 2.52 | 65.69 |
| L68-6 | 1131797400 | 1122173700 | 7481158 | 6969307 | 93.16 | 2.65 | 72.6 |
| L37-8 | 1002230100 | 974362800 | 6495752 | 6052394 | 93.17 | 2.46 | 67.72 |
| L75-4 | 1609372500 | 1594757700 | 10631718 | 9907740 | 93.19 | 3.43 | 80.18 |
| L59-9 | 1140310500 | 1128015900 | 7520106 | 7016741 | 93.31 | 2.65 | 72.79 |
| L35-10 | 1146811500 | 1138844700 | 7592298 | 7091884 | 93.41 | 2.75 | 73.57 |
| L39-5 | 1167992400 | 1148956200 | 7659708 | 7156915 | 93.44 | 2.76 | 73.84 |
| L8-2 | 1209237900 | 1197062700 | 7980418 | 7464862 | 93.54 | 2.68 | 71.76 |
| L74-8 | 1314034200 | 1299389700 | 8662598 | 8103206 | 93.54 | 2.96 | 76.83 |
| L21-7 | 1160891400 | 1137195300 | 7581302 | 7095208 | 93.59 | 2.68 | 73.18 |
| L68-10 | 1257779400 | 1217187300 | 8114582 | 7606645 | 93.74 | 2.72 | 73.13 |
| L28-4 | 1584973500 | 1552087800 | 10347252 | 9708741 | 93.83 | 3.42 | 79.61 |
| L40-9 | 1500727800 | 1472961000 | 9819740 | 9215813 | 93.85 | 3.24 | 79.12 |
| L13-9 | 1197594000 | 1181318400 | 7875456 | 7393538 | 93.88 | 2.81 | 73.15 |
| H13-2 | 1106728500 | 1086994200 | 7246628 | 6808408 | 93.95 | 2.64 | 73.03 |
| L59-8 | 1220311200 | 1197032100 | 7980214 | 7497818 | 93.96 | 2.89 | 73.92 |
| L74-6 | 1498488600 | 1479089100 | 9860594 | 9266258 | 93.97 | 3.3 | 77.9 |
| L18-4 | 1091609100 | 1075923000 | 7172820 | 6744180 | 94.02 | 2.62 | 72.1 |
| L31-1 | 1457371200 | 1441242900 | 9608286 | 9038840 | 94.07 | 3.14 | 79.04 |
| H34-9 | 825267300 | 791763900 | 5278426 | 4965865 | 94.08 | 2.26 | 62.51 |
| L21-2 | 1155249600 | 1130422200 | 7536148 | 7090745 | 94.09 | 2.75 | 73.02 |
| L69-10 | 808555800 | 790040400 | 5266936 | 4956727 | 94.11 | 2.15 | 65.81 |
| L21-3 | 1252017600 | 1225936500 | 8172910 | 7695740 | 94.16 | 2.85 | 75.51 |
| L21-5 | 1493982000 | 1459761900 | 9731746 | 9167566 | 94.2 | 3.27 | 78.11 |
| L2-1 | 1235500800 | 1219076400 | 8127176 | 7656587 | 94.21 | 2.83 | 75.35 |
| L42-2 | 1757763000 | 1728794700 | 11525298 | 10858193 | 94.21 | 3.68 | 81.63 |
| L110-6 | 1586415000 | 1555895400 | 10372636 | 9774932 | 94.24 | 3.37 | 77.56 |
| H74-24 | 1185987900 | 1151961000 | 7679740 | 7239177 | 94.26 | 2.76 | 72.57 |
| L68-7 | 1282059600 | 1243839900 | 8292266 | 7819817 | 94.3 | 2.88 | 74.53 |
| L52-9 | 1172226000 | 1142832600 | 7618884 | 7184793 | 94.3 | 2.75 | 73.81 |
| L6-25 | 1139069400 | 1106921700 | 7379478 | 6959678 | 94.31 | 2.66 | 71.42 |
| L6-4 | 1347939900 | 1326502500 | 8843350 | 8341877 | 94.33 | 3 | 77.79 |
| L8-22 | 1148968500 | 1120755300 | 7471702 | 7048611 | 94.34 | 2.7 | 71.79 |
| L61-1 | 1687927500 | 1631790000 | 10878600 | 10270858 | 94.41 | 3.56 | 78.81 |
| H6-3 | 1202524500 | 1185071700 | 7900478 | 7459653 | 94.42 | 2.79 | 73.88 |
| L39-6 | 1123054800 | 1106076300 | 7373842 | 6962222 | 94.42 | 2.72 | 73.11 |
| L36-5 | 1361630700 | 1352819700 | 9018798 | 8527089 | 94.55 | 3.08 | 78.65 |
| L59-6 | 1472181600 | 1440267600 | 9601784 | 9084072 | 94.61 | 3.27 | 78.15 |
| L52-3 | 1561144200 | 1533668100 | 10224454 | 9676134 | 94.64 | 3.36 | 80.11 |
| L36-9 | 1675934700 | 1658591400 | 11057276 | 10467692 | 94.67 | 3.47 | 81.75 |
| L21-4 | 1211014500 | 1180710600 | 7871404 | 7451937 | 94.67 | 2.79 | 73.29 |
| L44-6 | 1205643900 | 1193505300 | 7956702 | 7536924 | 94.72 | 2.85 | 75.15 |
| L3-8 | 1411955100 | 1383008700 | 9220058 | 8734198 | 94.73 | 3.11 | 77.6 |
| L82-8 | 1106212200 | 1076633700 | 7177558 | 6801093 | 94.75 | 2.65 | 72.6 |
| H44-1 | 1090967400 | 1068583500 | 7123890 | 6750619 | 94.76 | 2.61 | 72.47 |
| L41-7 | 1275977700 | 1251069300 | 8340462 | 7903857 | 94.77 | 2.94 | 75.68 |
| L109-53 | 1313760000 | 1276076400 | 8062863 | 8507176 | 94.78 | 2.89 | 74.4 |
| L37-6 | 1054392300 | 1041801600 | 6945344 | 6584388 | 94.8 | 2.55 | 70.49 |
| L80-2 | 1474727700 | 1454171700 | 9694478 | 9193581 | 94.83 | 3.2 | 78.82 |
| L37-4 | 1156635600 | 1121565900 | 7477106 | 7092165 | 94.85 | 2.79 | 71.64 |
| L87-9 | 1191927000 | 1161575100 | 7743834 | 7348894 | 94.9 | 2.75 | 73.74 |
| L46-9 | 1194744900 | 1177925400 | 7852836 | 7453868 | 94.92 | 2.8 | 75.03 |
| H68-2 | 993887400 | 977683800 | 6517892 | 6189092 | 94.96 | 2.51 | 69.4 |
| H36-3 | 1151915400 | 1129654800 | 7531032 | 7157469 | 95.04 | 2.72 | 74.55 |
| L81-2 | 1026580800 | 983012700 | 6553418 | 6229038 | 95.05 | 2.49 | 70.31 |
| L18-6 | 1591538700 | 1569734100 | 10464894 | 9952193 | 95.1 | 3.39 | 80.41 |
| L56-2 | 1208764500 | 1196634000 | 7977560 | 7587426 | 95.11 | 2.81 | 76.42 |
| L69-12 | 1310430000 | 1267361400 | 8449076 | 8036687 | 95.12 | 2.99 | 72.96 |
| L82-52 | 1184108700 | 1074302400 | 6813698 | 7162016 | 95.14 | 2.61 | 70.88 |
| L74-10 | 1292594100 | 1280018100 | 8533454 | 8121474 | 95.17 | 2.99 | 76.55 |
| L45-1 | 1079398200 | 1065093900 | 7100626 | 6759368 | 95.19 | 2.6 | 73.07 |
| H26-12 | 1096855500 | 1071468300 | 7143122 | 6799286 | 95.19 | 2.58 | 70.67 |
| L46-1 | 1188088500 | 1171965300 | 7813102 | 7437951 | 95.2 | 2.85 | 74.9 |
| L19-9 | 1124196000 | 1101192000 | 7341280 | 6989427 | 95.21 | 2.78 | 70.97 |
| L68-9 | 1407303900 | 1392808200 | 9285388 | 8846305 | 95.27 | 3.13 | 79.35 |
| L69-7 | 1424850000 | 1415559600 | 9437064 | 8994182 | 95.31 | 3.15 | 80.7 |
| L19-4 | 1017483600 | 1002534300 | 6683562 | 6372306 | 95.34 | 2.65 | 68.89 |
| L21-9 | 1053829200 | 1031391600 | 6875944 | 6556788 | 95.36 | 2.58 | 71.41 |
| L6-1 | 1210139700 | 1189763100 | 7931754 | 7564999 | 95.38 | 2.83 | 74.24 |
| H39-24 | 1102911000 | 1077637200 | 7184248 | 6853286 | 95.39 | 2.62 | 69.47 |
| L104-1 | 1352846100 | 1339557900 | 8930386 | 8521024 | 95.42 | 3.05 | 78.22 |
| L35-8 | 1359372900 | 1348766400 | 8991776 | 8581219 | 95.43 | 3.06 | 77.53 |
| L19-7 | 912087000 | 892861500 | 5952410 | 5684424 | 95.5 | 2.35 | 67.78 |
| L52-10 | 1236237000 | 1214186400 | 8094576 | 7730543 | 95.5 | 2.85 | 77.06 |
| L70-8 | 1065666900 | 1054507200 | 7030048 | 6716312 | 95.54 | 2.61 | 72.2 |
| H13-22 | 1215329700 | 1176059700 | 7840398 | 7492578 | 95.56 | 2.94 | 68.76 |
| L30-52 | 1330253700 | 1314758700 | 8375657 | 8765058 | 95.56 | 2.98 | 77.28 |
| L70-5 | 874355400 | 868528800 | 5790192 | 5533919 | 95.57 | 2.32 | 68.32 |
| L9-1 | 1321196100 | 1278910800 | 8526072 | 8150284 | 95.59 | 3.03 | 74.84 |
| L70-6 | 1321615800 | 1302290400 | 8681936 | 8299027 | 95.59 | 3.03 | 78.32 |
| H27-1 | 1134480000 | 1120555500 | 7470370 | 7141332 | 95.6 | 2.63 | 72.93 |
| H70-24 | 918870000 | 898094400 | 5987296 | 5723909 | 95.6 | 2.35 | 64.68 |
| L33-52 | 1578675900 | 1557087900 | 9925265 | 10380586 | 95.61 | 3.77 | 65.58 |
| H1-21 | 900313500 | 872364300 | 5815762 | 5560960 | 95.62 | 2.51 | 62.65 |
| L37-3 | 1194498000 | 1176789300 | 7845262 | 7501920 | 95.62 | 2.87 | 74.75 |
| L41-9 | 1063566000 | 1043948700 | 6959658 | 6655849 | 95.63 | 2.69 | 70.31 |
| L41-1 | 1241929200 | 1210566000 | 8070440 | 7719489 | 95.65 | 2.89 | 74.47 |
| L30-9 | 1259369700 | 1228588500 | 8190590 | 7834040 | 95.65 | 2.9 | 75.86 |
| L35-9 | 1343707800 | 1333750800 | 8891672 | 8504666 | 95.65 | 3.12 | 76.9 |
| L39-9 | 1040367900 | 1021854000 | 6812360 | 6517098 | 95.67 | 2.6 | 71.11 |
| L12-7 | 1751393400 | 1723544100 | 11490294 | 10994673 | 95.69 | 3.7 | 80.29 |
| H20-12 | 1130058000 | 1113007200 | 7420048 | 7100215 | 95.69 | 2.77 | 70.94 |
| H8-6 | 1297991700 | 1267547100 | 8450314 | 8086081 | 95.69 | 3.07 | 71.99 |
| L39-7 | 1664383800 | 1644818100 | 10965454 | 10495009 | 95.71 | 3.61 | 81.3 |
| L75-1 | 1853305200 | 1817605500 | 12117370 | 11598235 | 95.72 | 3.99 | 79.84 |
| L33-1 | 939153600 | 916675800 | 6111172 | 5850227 | 95.73 | 2.42 | 68.24 |
| L24-8 | 2024859600 | 1982552700 | 13217018 | 12652228 | 95.73 | 4.09 | 83.15 |
| H24-22 | 1001981400 | 984699900 | 6564666 | 6286319 | 95.76 | 2.57 | 67.75 |
| L64-22 | 970664100 | 940562100 | 6270414 | 6005641 | 95.78 | 2.55 | 65.8 |
| H2-47 | 943941300 | 923029800 | 6153532 | 5893933 | 95.78 | 2.49 | 66.44 |
| L42-11 | 1296593100 | 1261398600 | 8409324 | 8056358 | 95.8 | 2.93 | 75.21 |
| L36-7 | 1794026400 | 1776527700 | 11843518 | 11345838 | 95.8 | 3.73 | 81.93 |
| L79-8 | 1100379600 | 1079992500 | 7199950 | 6898213 | 95.81 | 2.62 | 72.16 |
| L81-11 | 1884304500 | 1844125200 | 12294168 | 11781050 | 95.83 | 3.89 | 80.95 |
| L40-1 | 1550340900 | 1519434600 | 10129564 | 9708751 | 95.85 | 3.45 | 79.55 |
| L52-6 | 1337538300 | 1314349200 | 8762328 | 8399312 | 95.86 | 3.08 | 77.07 |
| L43-1 | 1210640400 | 1183211700 | 7888078 | 7563134 | 95.88 | 2.85 | 74.54 |
| L34-52 | 1689525900 | 1661199300 | 10618652 | 11074662 | 95.88 | 3.57 | 79.05 |
| L52-7 | 1458750900 | 1433622000 | 9557480 | 9164514 | 95.89 | 3.29 | 78.79 |
| L18-7 | 1111358700 | 1087444500 | 7249630 | 6952107 | 95.9 | 2.67 | 71.24 |
| L52-1 | 1297502400 | 1284411300 | 8562742 | 8214694 | 95.94 | 2.98 | 77.09 |
| L70-4 | 1181957700 | 1172502000 | 7816680 | 7500079 | 95.95 | 2.76 | 76.76 |
| H39-21 | 1017807300 | 989355000 | 6595700 | 6328269 | 95.95 | 2.65 | 66.31 |
| L46-8 | 1356834000 | 1339258200 | 8928388 | 8569346 | 95.98 | 3.05 | 78.45 |
| L104-2 | 985478100 | 971787000 | 6478580 | 6219012 | 95.99 | 2.46 | 70.37 |
| L74-1 | 988539600 | 978430200 | 6522868 | 6262141 | 96 | 2.5 | 70.74 |
| H64-2 | 1501197600 | 1476593700 | 9843958 | 9450719 | 96.01 | 3.41 | 77.68 |
| H6-2 | 976928100 | 957934500 | 6386230 | 6131751 | 96.02 | 2.5 | 69.8 |
| L44-8 | 1173464100 | 1140303000 | 7602020 | 7300304 | 96.03 | 2.88 | 71.01 |
| L7-1 | 982394700 | 967538100 | 6450254 | 6195307 | 96.05 | 2.51 | 68.65 |
| L46-2 | 1147482000 | 1126407900 | 7509386 | 7213921 | 96.07 | 2.75 | 74.47 |
| H24-3 | 1185451800 | 1159407600 | 7729384 | 7425704 | 96.07 | 2.82 | 74.31 |
| H6-5 | 1521750600 | 1506561900 | 10043746 | 9652304 | 96.1 | 3.48 | 76.94 |
| H57-1 | 956800500 | 938873400 | 6259156 | 6014888 | 96.1 | 2.42 | 68.9 |
| L39-8 | 1087472100 | 1068102900 | 7120686 | 6843711 | 96.11 | 2.77 | 70.69 |
| L82-53 | 1635624900 | 1603571400 | 10274093 | 10690476 | 96.11 | 2.94 | 73.28 |
| H7-2 | 1238310900 | 1216108500 | 8107390 | 7792427 | 96.12 | 2.85 | 75.19 |
| H39-22 | 1083977400 | 1063023000 | 7086820 | 6812596 | 96.13 | 2.57 | 71.3 |
| L12-1 | 1134234300 | 1117089300 | 7447262 | 7159807 | 96.14 | 2.76 | 73.21 |
| L42-9 | 1477193100 | 1457204100 | 9714694 | 9339393 | 96.14 | 3.32 | 79.14 |
| L3-9 | 1130213400 | 1104368100 | 7362454 | 7079818 | 96.16 | 2.71 | 73.44 |
| H9-12 | 1487718600 | 1470026100 | 9800174 | 9425652 | 96.18 | 3.34 | 78.74 |
| L28-25 | 808923900 | 795467400 | 5303116 | 5100943 | 96.19 | 2.3 | 62.08 |
| L34-54 | 1811731200 | 1782794400 | 11433762 | 11885296 | 96.2 | 3.69 | 80.66 |
| H83-12 | 964785900 | 939298800 | 6261992 | 6023964 | 96.2 | 2.42 | 67.94 |
| L21-6 | 1402502100 | 1364084100 | 9093894 | 8749273 | 96.21 | 3.17 | 76.56 |
| L16-2 | 1355333400 | 1336379700 | 8909198 | 8573462 | 96.23 | 3.04 | 78.49 |
| L81-52 | 1713691200 | 1680683700 | 10781798 | 11204558 | 96.23 | 3.04 | 75.02 |
| L75-2 | 1512924000 | 1494420000 | 9962800 | 9588650 | 96.24 | 3.44 | 76.41 |
| H63-6 | 1168993200 | 1150001700 | 7666678 | 7378668 | 96.24 | 2.77 | 74.96 |
| L45-7 | 1352516700 | 1298398800 | 8655992 | 8332423 | 96.26 | 3.03 | 75.82 |
| L87-10 | 1409224500 | 1360124400 | 9067496 | 8729534 | 96.27 | 3.33 | 73.52 |
| H45-12 | 1429044000 | 1394537400 | 9296916 | 8949947 | 96.27 | 3.13 | 78.19 |
| L12-8 | 1168102500 | 1145712300 | 7638082 | 7354389 | 96.29 | 2.8 | 73.14 |
| H26-4 | 1268539500 | 1241435400 | 8276236 | 7970226 | 96.3 | 2.91 | 74.08 |
| H37-9 | 1448459400 | 1426307400 | 9508716 | 9157931 | 96.31 | 3.26 | 78.34 |
| L80-7 | 1020182700 | 1001598900 | 6677326 | 6431008 | 96.31 | 2.56 | 71.13 |
| L59-7 | 1483030800 | 1467276000 | 9781840 | 9421898 | 96.32 | 3.24 | 80.73 |
| L74-4 | 1127150700 | 1116861000 | 7445740 | 7172527 | 96.33 | 2.73 | 74.16 |
| L8-51 | 1490697900 | 1476425400 | 9482768 | 9842836 | 96.34 | 3.25 | 75.46 |
| H27-21 | 922817400 | 896381100 | 5975874 | 5757260 | 96.34 | 2.41 | 66.9 |
| H75-1 | 1116351600 | 1085713200 | 7238088 | 6974055 | 96.35 | 2.66 | 72.8 |
| L32-53 | 1534495800 | 1490612400 | 9574296 | 9937416 | 96.35 | 3.32 | 76.41 |
| H20-4 | 1370243700 | 1329625200 | 8864168 | 8540386 | 96.35 | 3.15 | 75.26 |
| L87-1 | 1135792500 | 1102473900 | 7349826 | 7082498 | 96.36 | 2.69 | 73.87 |
| H81-11 | 1001167500 | 975659400 | 6504396 | 6267929 | 96.36 | 2.53 | 68.71 |
| L87-2 | 1710625800 | 1680250200 | 11201668 | 10793454 | 96.36 | 3.63 | 80.12 |
| H66-1 | 1164052500 | 1151013600 | 7673424 | 7394940 | 96.37 | 2.77 | 73.58 |
| L17-57 | 1304263500 | 1244115000 | 7993186 | 8294100 | 96.37 | 2.96 | 73.58 |
| L61-51 | 1660593000 | 1629341100 | 10469974 | 10862274 | 96.39 | 3.42 | 78.25 |
| L74-7 | 1249104600 | 1238533200 | 8256888 | 7959014 | 96.39 | 2.89 | 75.85 |
| H73-11 | 1060335600 | 1034577600 | 6897184 | 6650238 | 96.42 | 2.6 | 70.53 |
| L30-51 | 1342212000 | 1277327400 | 8210542 | 8515516 | 96.42 | 3.04 | 73.22 |
| L110-10 | 1305346800 | 1276502100 | 8510014 | 8206363 | 96.43 | 2.98 | 76.22 |
| L6-2 | 1118258700 | 1101561300 | 7343742 | 7081425 | 96.43 | 2.71 | 73 |
| L44-13 | 1260643800 | 1231965000 | 8213100 | 7920729 | 96.44 | 2.95 | 73.58 |
| L64-16 | 1281151500 | 1243607100 | 8290714 | 7995336 | 96.44 | 2.92 | 73.3 |
| L70-51 | 1660575900 | 1268016600 | 8152979 | 8453444 | 96.45 | 2.89 | 76.41 |
| H27-22 | 990719700 | 966594300 | 6443962 | 6215887 | 96.46 | 2.47 | 68.1 |
| L61-54 | 1896131700 | 1862230200 | 11978458 | 12414868 | 96.48 | 3.22 | 77.31 |
| H14-21 | 948192000 | 909025200 | 6060168 | 5847580 | 96.49 | 2.57 | 63.03 |
| H40-11 | 1421004300 | 1380624300 | 9204162 | 8881246 | 96.49 | 3.13 | 77.33 |
| L6-5 | 1081630500 | 1060991100 | 7073274 | 6825745 | 96.5 | 2.68 | 70.44 |
| H26-23 | 1126960500 | 989728800 | 6598192 | 6367282 | 96.5 | 2.68 | 65.69 |
| L28-51 | 1342655400 | 1275083400 | 8203522 | 8500556 | 96.51 | 3 | 73.27 |
| H42-1 | 1332976200 | 1310400300 | 8736002 | 8431577 | 96.52 | 3.1 | 77.22 |
| H29-1 | 2032086600 | 2002763400 | 13351756 | 12886615 | 96.52 | 4.24 | 84.06 |
| H10-21 | 952363800 | 928169400 | 6187796 | 5972500 | 96.52 | 2.53 | 65.95 |
| L80-51 | 1660749000 | 1617476700 | 10407798 | 10783178 | 96.52 | 2.99 | 74.67 |
| H77-23 | 1116164400 | 1096512000 | 7310080 | 7055548 | 96.52 | 2.69 | 72.44 |
| H74-1 | 1122576900 | 1098639300 | 7324262 | 7070042 | 96.53 | 2.76 | 70.79 |
| L9-53 | 1279778400 | 1263628500 | 8131913 | 8424190 | 96.53 | 2.98 | 74.89 |
| H1-22 | 877760400 | 865223100 | 5768154 | 5568475 | 96.54 | 2.36 | 67.21 |
| L19-6 | 1075196400 | 1054890300 | 7032602 | 6789220 | 96.54 | 2.63 | 72.13 |
| L6-51 | 1800744000 | 1779644700 | 11454263 | 11864298 | 96.54 | 3.96 | 75.59 |
| L30-1 | 1338153000 | 1311472800 | 8743152 | 8441163 | 96.55 | 3.1 | 77.03 |
| H49-23 | 1076929500 | 1057316100 | 7048774 | 6805553 | 96.55 | 2.64 | 69.54 |
| H36-41 | 1009558500 | 983001300 | 6553342 | 6327563 | 96.55 | 2.55 | 68.55 |
| H79-1 | 1110615900 | 1079837100 | 7198914 | 6951232 | 96.56 | 2.76 | 70.45 |
| L24-6 | 1935434700 | 1903229100 | 12688194 | 12251613 | 96.56 | 4.05 | 83.68 |
| L32-55 | 1429341000 | 1408334700 | 9065494 | 9388898 | 96.56 | 3.27 | 74.68 |
| H1-1 | 1529112600 | 1514996700 | 10099978 | 9753638 | 96.57 | 3.36 | 80.47 |
| L74-55 | 1627715400 | 1414364400 | 9105781 | 9429096 | 96.57 | 3.14 | 77.37 |
| H14-9 | 1354826400 | 1337941200 | 8919608 | 8614437 | 96.58 | 3.07 | 77.75 |
| L82-1 | 1363378200 | 1329778800 | 8865192 | 8563557 | 96.6 | 3.21 | 75.85 |
| L36-1 | 1008425100 | 1000698300 | 6671322 | 6444382 | 96.6 | 2.55 | 71.88 |
| H6-8 | 1179517200 | 1146588600 | 7643924 | 7383790 | 96.6 | 2.79 | 72.83 |
| L68-52 | 1836063600 | 1411274400 | 9089297 | 9408496 | 96.61 | 3.2 | 77.92 |
| L32-1 | 1135299900 | 1116305700 | 7442038 | 7190845 | 96.62 | 2.77 | 70.48 |
| L44-21 | 1076819100 | 1045528200 | 6970188 | 6734698 | 96.62 | 2.66 | 70.17 |
| L19-5 | 1260169200 | 1237423500 | 8249490 | 7970352 | 96.62 | 2.91 | 76.27 |
| H4-11 | 1500043200 | 1454145000 | 9694300 | 9366437 | 96.62 | 3.27 | 78.47 |
| H57-10 | 1061031300 | 1046295300 | 6975302 | 6740257 | 96.63 | 2.63 | 72.15 |
| L6-53 | 2045814300 | 2024618700 | 13042498 | 13497458 | 96.63 | 4.13 | 81.27 |
| H39-8 | 1317957600 | 1295717100 | 8638114 | 8347973 | 96.64 | 2.98 | 77.53 |
| L82-54 | 1718622300 | 1682727900 | 10841092 | 11218186 | 96.64 | 3.05 | 74.53 |
| H24-8 | 1088945100 | 1049571600 | 6997144 | 6762176 | 96.64 | 2.62 | 72.06 |
| L47-8 | 1135437000 | 1119757800 | 7465052 | 7214699 | 96.65 | 2.74 | 74.31 |
| L28-52 | 1419181200 | 1358985900 | 8757397 | 9059906 | 96.66 | 3.14 | 74.8 |
| L3-7 | 1877749800 | 1840822200 | 12272148 | 11862096 | 96.66 | 4.05 | 82.73 |
| H64-4 | 1426784100 | 1399807200 | 9332048 | 9021149 | 96.67 | 3.25 | 78.76 |
| L80-52 | 1604811000 | 1507042500 | 9712758 | 10046950 | 96.67 | 3.24 | 78.24 |
| L79-5 | 1148426400 | 1130419200 | 7536128 | 7285741 | 96.68 | 2.71 | 73.86 |
| L32-56 | 1755748200 | 1742891100 | 11233490 | 11619274 | 96.68 | 4.14 | 70.09 |
| H9-22 | 1199008800 | 1178334300 | 7855562 | 7594447 | 96.68 | 2.83 | 72.96 |
| L43-51 | 1935164100 | 1912277400 | 12328494 | 12748516 | 96.71 | 3.79 | 76.1 |
| H44-24 | 985269600 | 963219900 | 6421466 | 6210320 | 96.71 | 2.46 | 67.22 |
| L32-54 | 1678639500 | 1655763000 | 10675274 | 11038420 | 96.71 | 3.96 | 69.1 |
| L9-51 | 1575489600 | 1556811600 | 10038929 | 10378744 | 96.73 | 3.42 | 76.81 |
| H47-3 | 1924006500 | 1881298800 | 12541992 | 12133466 | 96.74 | 3.93 | 82.59 |
| L92-9 | 1406895300 | 1358898600 | 9059324 | 8763651 | 96.74 | 3.23 | 76.31 |
| L37-7 | 1390488600 | 1353682200 | 9024548 | 8730102 | 96.74 | 3.15 | 76.74 |
| L53-10 | 1006544400 | 993505800 | 6623372 | 6408020 | 96.75 | 2.53 | 70.53 |
| L18-52 | 1442571900 | 1354447800 | 8736526 | 9029652 | 96.75 | 3.1 | 74.68 |
| L81-8 | 1291468500 | 1272475200 | 8483168 | 8208500 | 96.76 | 2.99 | 77.06 |
| H63-23 | 1246454700 | 1216966200 | 8113108 | 7850443 | 96.76 | 2.93 | 73.62 |
| H4-4 | 1423134000 | 1375103700 | 9167358 | 8871178 | 96.77 | 3.16 | 78.19 |
| H50-14 | 1961916900 | 1923306900 | 12822046 | 12409333 | 96.78 | 3.9 | 84.45 |
| H45-21 | 1246764600 | 1223026500 | 8153510 | 7892096 | 96.79 | 2.88 | 73.91 |
| H83-24 | 1279271100 | 1254009000 | 8360060 | 8091715 | 96.79 | 2.96 | 75.62 |
| H47-22 | 967122900 | 944018700 | 6293458 | 6092091 | 96.8 | 2.51 | 67.48 |
| L24-5 | 1764264300 | 1737837000 | 11585580 | 11214606 | 96.8 | 3.73 | 82.66 |
| L110-52 | 1879748400 | 1845971700 | 11913033 | 12306478 | 96.8 | 3.76 | 81.97 |
| H7-23 | 1050213000 | 1023472200 | 6823148 | 6604693 | 96.8 | 2.61 | 70.28 |
| H50-41 | 1070125500 | 1051939200 | 7012928 | 6788778 | 96.8 | 2.73 | 69.41 |
| L28-11 | 1335007800 | 1292549700 | 8616998 | 8342050 | 96.81 | 2.99 | 77.79 |
| H44-5 | 1207953600 | 1182189900 | 7881266 | 7629878 | 96.81 | 2.79 | 74.42 |
| L39-10 | 1414554000 | 1391007300 | 9273382 | 8977872 | 96.81 | 3.17 | 78.04 |
| H69-47 | 949107000 | 920785800 | 6138572 | 5942637 | 96.81 | 2.43 | 68.09 |
| H25-13 | 1279591500 | 1242255600 | 8281704 | 8017465 | 96.81 | 2.93 | 73.26 |
| H79-2 | 1422541200 | 1389787800 | 9265252 | 8971024 | 96.82 | 3.11 | 78.26 |
| L13-2 | 1234288800 | 1222746300 | 8151642 | 7892593 | 96.82 | 2.89 | 76.46 |
| H35-10 | 1357959000 | 1332883800 | 8885892 | 8603479 | 96.82 | 2.86 | 73.59 |
| H2-22 | 987716700 | 973464900 | 6489766 | 6283163 | 96.82 | 2.49 | 69.14 |
| L16-1 | 1234055100 | 1212483900 | 8083226 | 7826875 | 96.83 | 2.83 | 76.91 |
| H70-1 | 1151579700 | 1126161600 | 7507744 | 7269655 | 96.83 | 2.79 | 73.61 |
| L79-51 | 1573586100 | 1502316300 | 9697836 | 10015442 | 96.83 | 3.19 | 78.25 |
| L18-53 | 1300595100 | 1261165200 | 8141231 | 8407768 | 96.83 | 3.03 | 73.73 |
| L14-1 | 1227636900 | 1213934700 | 8092898 | 7837021 | 96.84 | 2.82 | 75.53 |
| L14-51 | 1447380300 | 1426053300 | 9206223 | 9507022 | 96.84 | 3.19 | 76 |
| H39-23 | 931103400 | 909565500 | 6063770 | 5871971 | 96.84 | 2.55 | 64.89 |
| L21-52 | 1486630200 | 1435870500 | 9270428 | 9572470 | 96.84 | 3.25 | 76.4 |
| H11-2 | 877505700 | 864131700 | 5760878 | 5579290 | 96.85 | 2.33 | 67.17 |
| H81-7 | 1468524300 | 1442392800 | 9615952 | 9313268 | 96.85 | 3.27 | 79.24 |
| L8-1 | 1199126700 | 1168422000 | 7789480 | 7545070 | 96.86 | 2.84 | 73.37 |
| L41-6 | 1400834700 | 1386335400 | 9242236 | 8952126 | 96.86 | 3.17 | 79.45 |
| L6-54 | 2051479800 | 2023054500 | 13063890 | 13487030 | 96.86 | 4.34 | 78.37 |
| L47-2 | 1292298900 | 1254147900 | 8360986 | 8099593 | 96.87 | 2.99 | 76.81 |
| H31-4 | 1109922900 | 1093418700 | 7289458 | 7062333 | 96.88 | 2.73 | 72.45 |
| H63-13 | 1649764500 | 1607057400 | 10713716 | 10379479 | 96.88 | 3.49 | 80.66 |
| L6-52 | 1647402900 | 1624532400 | 10493337 | 10830216 | 96.89 | 3.56 | 77.94 |
| H69-46 | 991776000 | 964904100 | 6432694 | 6232795 | 96.89 | 2.53 | 67.35 |
| H38-7 | 1066240200 | 1025144700 | 6834298 | 6622325 | 96.9 | 2.63 | 70.45 |
| L9-52 | 1597952400 | 1573814100 | 10167120 | 10492094 | 96.9 | 3.4 | 77.84 |
| L18-51 | 1311408900 | 1289102100 | 8327313 | 8594014 | 96.9 | 3 | 74.65 |
| H50-21 | 1078397400 | 1055117700 | 7034118 | 6816091 | 96.9 | 2.65 | 70.73 |
| L12-9 | 1147296900 | 1120197600 | 7467984 | 7236894 | 96.91 | 2.89 | 69.76 |
| H7-3 | 1055308800 | 1024130700 | 6827538 | 6616492 | 96.91 | 2.66 | 70.1 |
| H65-11 | 1826877300 | 1783090800 | 11887272 | 11521266 | 96.92 | 3.72 | 82.19 |
| H50-42 | 876833400 | 859589700 | 5730598 | 5553895 | 96.92 | 2.34 | 66.11 |
| H19-2 | 995781600 | 985327500 | 6568850 | 6367212 | 96.93 | 2.67 | 66.73 |
| H63-45 | 980738400 | 958743900 | 6391626 | 6195205 | 96.93 | 2.52 | 68.61 |
| H38-1 | 1546294500 | 1521954600 | 10146364 | 9836254 | 96.94 | 3.38 | 80.56 |
| L47-10 | 1116927300 | 1100529000 | 7336860 | 7113238 | 96.95 | 2.65 | 73.49 |
| L12-10 | 1078279200 | 1037866800 | 6919112 | 6708345 | 96.95 | 2.7 | 69.42 |
| H9-5 | 1165323300 | 1133066700 | 7553778 | 7323285 | 96.95 | 2.83 | 72.89 |
| L17-56 | 1205593800 | 1172526300 | 7579560 | 7816842 | 96.96 | 2.8 | 72.94 |
| L45-6 | 1697671800 | 1676506500 | 11176710 | 10838472 | 96.97 | 3.66 | 81.44 |
| H25-21 | 1175760300 | 1069616400 | 7130776 | 6914965 | 96.97 | 2.59 | 68.34 |
| H77-26 | 1406218500 | 1377871200 | 9185808 | 8907040 | 96.97 | 3.17 | 77.27 |
| L21-51 | 1439832300 | 1383886800 | 8946990 | 9225912 | 96.98 | 3.18 | 76.19 |
| L9-54 | 1586931600 | 1563492600 | 10109692 | 10423284 | 96.99 | 3.38 | 78.19 |
| H24-12 | 1141332900 | 1115747400 | 7438316 | 7214659 | 96.99 | 2.62 | 72.39 |
| L33-8 | 1562277000 | 1522671300 | 10151142 | 9846851 | 97 | 3.43 | 78.61 |
| H68-1 | 1460859900 | 1437323400 | 9582156 | 9295630 | 97.01 | 3.26 | 78.4 |
| L74-51 | 1237121400 | 1215315600 | 7859582 | 8102104 | 97.01 | 2.95 | 73.22 |
| L13-1 | 1488681600 | 1463308800 | 9755392 | 9464670 | 97.02 | 3.22 | 77.75 |
| L109-51 | 1475283000 | 1454687100 | 9408621 | 9697914 | 97.02 | 3.32 | 76.53 |
| L45-55 | 1626895500 | 1600467300 | 10351714 | 10669782 | 97.02 | 3.42 | 79.31 |
| L34-56 | 1668157800 | 1656500700 | 10714766 | 11043338 | 97.02 | 3.96 | 68.06 |
| H65-2 | 1341093000 | 1308499200 | 8723328 | 8463034 | 97.02 | 3 | 76.99 |
| H14-2 | 1086168900 | 1068317700 | 7122118 | 6910422 | 97.03 | 2.66 | 71.37 |
| H35-2 | 1282100700 | 1263470400 | 8423136 | 8172663 | 97.03 | 2.92 | 76.7 |
| H65-3 | 1144959000 | 1102927500 | 7352850 | 7134502 | 97.03 | 2.85 | 69.4 |
| L87-8 | 1111160700 | 1089414300 | 7262762 | 7047513 | 97.04 | 2.7 | 73.06 |
| L61-52 | 1437408300 | 1351141500 | 8741531 | 9007610 | 97.05 | 3.06 | 76.4 |
| L17-51 | 1397223000 | 1373484000 | 8886789 | 9156560 | 97.05 | 3.18 | 76.78 |
| H77-24 | 1442001600 | 1412569500 | 9417130 | 9139456 | 97.05 | 3.18 | 78.25 |
| L44-22 | 931987800 | 910549500 | 6070330 | 5892014 | 97.06 | 2.46 | 66.82 |
| H31-3 | 1165607400 | 1140361800 | 7602412 | 7379303 | 97.07 | 2.73 | 70.09 |
| L79-1 | 1236685500 | 1213659000 | 8091060 | 7854286 | 97.07 | 2.94 | 75.45 |
| H10-1 | 1531515300 | 1505304300 | 10035362 | 9742731 | 97.08 | 3.44 | 79.51 |
| L64-1 | 1305638700 | 1254926100 | 8366174 | 8122389 | 97.09 | 3.01 | 74.86 |
| L43-52 | 1967871900 | 1950764100 | 12626854 | 13005094 | 97.09 | 3.85 | 77.58 |
| L28-54 | 1536054600 | 1491441900 | 9653559 | 9942946 | 97.09 | 3.28 | 77.23 |
| L74-53 | 1621745100 | 1599936000 | 10356717 | 10666240 | 97.1 | 3.57 | 80.87 |
| L18-5 | 948684000 | 921390000 | 6142600 | 5965308 | 97.11 | 2.52 | 66.85 |
| L92-23 | 883566000 | 856854900 | 5712366 | 5547426 | 97.11 | 2.41 | 64.33 |
| L2-52 | 1788740700 | 1762551600 | 11411296 | 11750344 | 97.11 | 3.79 | 78.02 |
| H27-8 | 1052936400 | 1029333900 | 6862226 | 6663888 | 97.11 | 2.58 | 71.37 |
| H3-21 | 1421656500 | 1399498800 | 9329992 | 9060813 | 97.11 | 3.2 | 77.14 |
| H7-5 | 1581566700 | 1554976200 | 10366508 | 10067161 | 97.11 | 3.47 | 78.89 |
| L110-51 | 1566481800 | 1539567000 | 9968216 | 10263780 | 97.12 | 3.36 | 79.34 |
| L82-55 | 1774789800 | 1744483500 | 11294584 | 11629890 | 97.12 | 3.7 | 81.76 |
| H83-4 | 1295667600 | 1268322600 | 8455484 | 8211872 | 97.12 | 2.95 | 76.62 |
| H62-10 | 1138028100 | 1119593700 | 7463958 | 7249866 | 97.13 | 2.75 | 72.64 |
| L45-51 | 1727852400 | 1708762500 | 11066142 | 11391750 | 97.14 | 3.68 | 77.69 |
| H24-11 | 1190775300 | 1153111200 | 7687408 | 7467767 | 97.14 | 2.86 | 72.27 |
| H43-1 | 1300771500 | 1279994100 | 8533294 | 8290365 | 97.15 | 2.98 | 77.16 |
| H34-8 | 1194181800 | 1178758800 | 7858392 | 7634482 | 97.15 | 2.86 | 74.93 |
| L61-56 | 1498881000 | 1148881500 | 7441113 | 7659210 | 97.15 | 2.8 | 73.53 |
| L32-57 | 1421758200 | 1403751300 | 9092687 | 9358342 | 97.16 | 3.64 | 64.97 |
| L32-52 | 1339281600 | 1282422600 | 8306493 | 8549484 | 97.16 | 3.06 | 73.43 |
| L33-51 | 1602339300 | 1580815500 | 10239616 | 10538770 | 97.16 | 3.6 | 75.32 |
| L17-53 | 1496765100 | 1468134000 | 9509566 | 9787560 | 97.16 | 3.32 | 77.59 |
| H63-2 | 1220301600 | 1206020700 | 8040138 | 7812090 | 97.16 | 2.89 | 76.2 |
| H36-26 | 1238386500 | 1208873100 | 8059154 | 7830486 | 97.16 | 2.83 | 73.75 |
| H50-26 | 1400552700 | 1365784800 | 9105232 | 8846192 | 97.16 | 3.12 | 77.03 |
| L34-51 | 1648892400 | 1635247800 | 10593376 | 10901652 | 97.17 | 3.89 | 68.11 |
| L61-53 | 1444498200 | 1423455300 | 9221703 | 9489702 | 97.18 | 3.28 | 78.46 |
| H9-4 | 930283800 | 913747800 | 6091652 | 5919711 | 97.18 | 2.49 | 68.18 |
| H4-12 | 1395315300 | 1365997200 | 9106648 | 8849792 | 97.18 | 3.23 | 76.34 |
| H36-23 | 1268050800 | 1206450900 | 8043006 | 7816682 | 97.19 | 3.1 | 68.99 |
| H83-25 | 1102042800 | 1076022300 | 7173482 | 6971957 | 97.19 | 2.68 | 71.49 |
| L61-57 | 1537383000 | 1181579700 | 7656805 | 7877198 | 97.2 | 2.84 | 74.57 |
| H39-9 | 1306362900 | 1284932400 | 8566216 | 8327548 | 97.21 | 3.05 | 77.92 |
| L47-1 | 1438253100 | 1422622200 | 9484148 | 9220686 | 97.22 | 3.27 | 78.65 |
| H68-21 | 1082858700 | 1068798300 | 7125322 | 6928003 | 97.23 | 2.74 | 71.3 |
| H74-7 | 1208224800 | 1182591000 | 7883940 | 7665833 | 97.23 | 2.89 | 74.78 |
| L39-4 | 1004244900 | 982727100 | 6551514 | 6370332 | 97.23 | 2.5 | 70.67 |
| L61-55 | 1732156500 | 1326320700 | 8597427 | 8842138 | 97.23 | 3.11 | 75.83 |
| H42-2 | 1663801500 | 1651373400 | 11009156 | 10705776 | 97.24 | 3.7 | 80.54 |
| L19-51 | 1718916300 | 1689735600 | 10954118 | 11264904 | 97.24 | 3.63 | 80.23 |
| L34-57 | 1792908000 | 1781873400 | 11553029 | 11879156 | 97.25 | 3.68 | 74.54 |
| H10-2 | 1544121300 | 1518078600 | 10120524 | 9842891 | 97.26 | 3.55 | 75.8 |
| L14-53 | 1438617300 | 1417962000 | 9193932 | 9453080 | 97.26 | 3.22 | 78.77 |
| H9-3 | 1362866400 | 1343642100 | 8957614 | 8712618 | 97.26 | 3.16 | 77.74 |
| H8-22 | 1031134800 | 1007945400 | 6719636 | 6535542 | 97.26 | 2.48 | 67.78 |
| L28-53 | 1961765100 | 1930654800 | 12519016 | 12871032 | 97.27 | 4.1 | 80.98 |
| H63-12 | 1441242900 | 1417254300 | 9448362 | 9190452 | 97.27 | 3.28 | 78.32 |
| H76-23 | 1077319200 | 1062052800 | 7080352 | 6887445 | 97.28 | 2.67 | 71.72 |
| L30-6 | 1145181000 | 1127945400 | 7519636 | 7315382 | 97.28 | 2.77 | 74.48 |
| L17-54 | 1388989800 | 1368532200 | 8875411 | 9123548 | 97.28 | 3.2 | 76.23 |
| H8-2 | 1380227700 | 1355653500 | 9037690 | 8791496 | 97.28 | 3.02 | 79.12 |
| L17-55 | 1275303900 | 1254817200 | 8138585 | 8365448 | 97.29 | 2.99 | 74.66 |
| H69-21 | 1143958200 | 1113774600 | 7425164 | 7223807 | 97.29 | 2.72 | 72.83 |
| H13-1 | 1400325000 | 1344214800 | 8961432 | 8719035 | 97.3 | 3.12 | 75.03 |
| H31-2 | 1146789300 | 1102026000 | 7346840 | 7148393 | 97.3 | 2.66 | 68.17 |
| H70-23 | 1297684500 | 1269129600 | 8460864 | 8232446 | 97.3 | 2.87 | 73.55 |
| H3-3 | 1274906400 | 1246356900 | 8309046 | 8085014 | 97.3 | 2.78 | 75.47 |
| H36-4 | 1444855200 | 1416131700 | 9440878 | 9186451 | 97.31 | 3.26 | 78.54 |
| H36-24 | 1217841900 | 1168486800 | 7789912 | 7580307 | 97.31 | 2.74 | 70.97 |
| H24-23 | 1151574900 | 1136829900 | 7578866 | 7375930 | 97.32 | 2.82 | 72.45 |
| L17-1 | 1234999500 | 1218214500 | 8121430 | 7904809 | 97.33 | 2.78 | 73.16 |
| H3-4 | 1437505200 | 1412246100 | 9414974 | 9163607 | 97.33 | 3.13 | 78.28 |
| H31-1 | 1075614900 | 1049747700 | 6998318 | 6812868 | 97.35 | 2.55 | 69.07 |
| L92-1 | 1253279700 | 1234559100 | 8230394 | 8012735 | 97.36 | 2.93 | 76.16 |
| L59-1 | 1032397500 | 1017395400 | 6782636 | 6603515 | 97.36 | 2.63 | 71.56 |
| H70-25 | 954676200 | 934102200 | 6227348 | 6062678 | 97.36 | 2.48 | 67.94 |
| L109-52 | 1925027700 | 1889354100 | 12264510 | 12595694 | 97.37 | 3.9 | 82.13 |
| L74-54 | 1836690000 | 1819166100 | 11809164 | 12127774 | 97.37 | 3.91 | 83.62 |
| H24-21 | 1177374900 | 1155873300 | 7705822 | 7503213 | 97.37 | 2.74 | 70.9 |
| H6-1 | 1252724100 | 1235114100 | 8234094 | 8018665 | 97.38 | 3.05 | 73.18 |
| L81-51 | 1526732400 | 1503746700 | 9762101 | 10024978 | 97.38 | 3.4 | 78.59 |
| L92-52 | 1777141500 | 1747580700 | 11344894 | 11650538 | 97.38 | 3.59 | 82.84 |
| H19-1 | 1617125700 | 1593846000 | 10625640 | 10348672 | 97.39 | 3.56 | 79.63 |
| L37-52 | 2250978600 | 2223702300 | 14437701 | 14824682 | 97.39 | 4.54 | 79.51 |
| L17-52 | 1505184600 | 1472948400 | 9563157 | 9819656 | 97.39 | 3.38 | 77.2 |
| H26-24 | 1047217500 | 949472100 | 6329814 | 6164764 | 97.39 | 2.62 | 65.49 |
| L70-7 | 1434436500 | 1417539600 | 9450264 | 9204912 | 97.4 | 3.21 | 79.76 |
| H64-3 | 1860157500 | 1834612200 | 12230748 | 11913908 | 97.41 | 3.95 | 82.76 |
| L14-6 | 975273000 | 966143100 | 6440954 | 6274153 | 97.41 | 2.45 | 70.68 |
| L47-9 | 1201590300 | 1181140200 | 7874268 | 7670726 | 97.42 | 2.91 | 74.32 |
| L44-7 | 1540243200 | 1506037800 | 10040252 | 9781016 | 97.42 | 3.41 | 78.6 |
| L110-54 | 1858667700 | 1825767900 | 11857444 | 12171786 | 97.42 | 3.72 | 83.21 |
| H26-5 | 1088026800 | 1069338000 | 7128920 | 6944721 | 97.42 | 2.67 | 73.12 |
| L40-51 | 1776300300 | 1763263800 | 11452485 | 11755092 | 97.43 | 3.8 | 75.54 |
| L30-22 | 1208287800 | 1190406000 | 7936040 | 7732074 | 97.43 | 2.89 | 74.67 |
| L41-51 | 1290514200 | 1270703100 | 8253955 | 8471354 | 97.43 | 2.98 | 76.34 |
| L109-57 | 2010393000 | 1968003600 | 12782658 | 13120024 | 97.43 | 4.05 | 82.96 |
| L34-55 | 1287954900 | 1269297000 | 8244445 | 8461980 | 97.43 | 3 | 75.71 |
| H47-1 | 1386594900 | 1343190300 | 8954602 | 8725561 | 97.44 | 3.01 | 74.13 |
| L74-52 | 1427249700 | 1406646600 | 9137689 | 9377644 | 97.44 | 3.21 | 77.64 |
| H50-13 | 1212430500 | 1190176200 | 7934508 | 7731586 | 97.44 | 2.83 | 75.6 |
| H4-21 | 1065063300 | 1038304200 | 6922028 | 6744917 | 97.44 | 2.75 | 68.05 |
| L41-52 | 1511118900 | 1490927400 | 9686125 | 9939516 | 97.45 | 3.43 | 77.7 |
| L32-51 | 1257550200 | 1144872000 | 7438116 | 7632480 | 97.45 | 3.16 | 62.84 |
| H20-3 | 1083234600 | 1072160100 | 7147734 | 6965258 | 97.45 | 2.66 | 73.59 |
| L39-1 | 1138106400 | 1110661500 | 7404410 | 7216069 | 97.46 | 2.79 | 73.24 |
| L34-53 | 1415413200 | 1396858800 | 9075910 | 9312392 | 97.46 | 3.23 | 76.08 |
| H45-23 | 1220255700 | 1196223300 | 7974822 | 7772249 | 97.46 | 2.84 | 74.38 |
| L35-1 | 1107884700 | 1099349100 | 7328994 | 7143349 | 97.47 | 2.73 | 73.83 |
| H45-14 | 1434469800 | 1401906000 | 9346040 | 9109297 | 97.47 | 3.25 | 77.5 |
| H45-41 | 1122523500 | 1098494700 | 7323298 | 7137948 | 97.47 | 2.62 | 71.85 |
| L14-52 | 1543572900 | 1519836600 | 9876446 | 10132244 | 97.48 | 3.41 | 78.8 |
| L68-51 | 2031717600 | 1551566700 | 10083044 | 10343778 | 97.48 | 3.48 | 78.15 |
| H65-13 | 1279547400 | 1255821900 | 8372146 | 8162000 | 97.49 | 2.95 | 76.64 |
| H8-24 | 1210616400 | 1190809200 | 7938728 | 7739463 | 97.49 | 2.74 | 72.64 |
| H62-1 | 1566005700 | 1538028000 | 10253520 | 9996792 | 97.5 | 3.51 | 80.52 |
| H8-4 | 1797322200 | 1749173400 | 11661156 | 11369733 | 97.5 | 3.71 | 82.85 |
| L42-51 | 1373187900 | 1353479100 | 8798535 | 9023194 | 97.51 | 3.21 | 75.22 |
| H11-1 | 1060602900 | 1042032300 | 6946882 | 6774524 | 97.52 | 2.61 | 70.24 |
| L37-51 | 1463663400 | 1445681400 | 9398875 | 9637876 | 97.52 | 3.3 | 78.28 |
| H57-21 | 918454800 | 904052100 | 6027014 | 5877956 | 97.53 | 2.47 | 66.88 |
| H27-9 | 937840500 | 885343500 | 5902290 | 5756288 | 97.53 | 2.38 | 67.78 |
| H7-4 | 1177546800 | 1151634000 | 7677560 | 7488649 | 97.54 | 2.85 | 73.72 |
| H39-10 | 1857594300 | 1820979600 | 12139864 | 11845464 | 97.57 | 3.56 | 79.3 |
| L45-52 | 1607077800 | 1598046300 | 10394643 | 10653642 | 97.57 | 3.63 | 74.98 |
| L92-51 | 2064474900 | 2018926800 | 13134408 | 13459512 | 97.58 | 4.02 | 83.66 |
| L110-53 | 1696317900 | 1638604200 | 10660172 | 10924028 | 97.58 | 3.58 | 79.99 |
| H8-5 | 1186135800 | 1168117800 | 7787452 | 7600114 | 97.59 | 2.95 | 72.06 |
| L44-52 | 1460292000 | 1439148000 | 9364246 | 9594320 | 97.6 | 3.35 | 76.65 |
| L45-53 | 1869652500 | 1857309900 | 12088593 | 12382066 | 97.63 | 3.93 | 77.57 |
| H20-6 | 1520252700 | 1482758400 | 9885056 | 9650826 | 97.63 | 3.38 | 80.08 |
| L110-9 | 1287591600 | 1242135000 | 8280900 | 8085645 | 97.64 | 3.08 | 74.42 |
| H19-3 | 950562900 | 940071300 | 6267142 | 6119764 | 97.65 | 2.47 | 69.11 |
| L109-55 | 1840780500 | 1796196300 | 11693386 | 11974642 | 97.65 | 3.82 | 80.49 |
| H36-21 | 1065330300 | 1031354100 | 6875694 | 6714275 | 97.65 | 2.64 | 68.68 |
| L70-1 | 976929300 | 969370500 | 6462470 | 6311116 | 97.66 | 2.49 | 71.78 |
| L45-54 | 1276304400 | 1127635200 | 7341718 | 7517568 | 97.66 | 2.73 | 73.22 |
| L68-1 | 1252506900 | 1193020500 | 7953470 | 7769812 | 97.69 | 2.9 | 75.74 |
| L44-51 | 1442325000 | 1421856000 | 9260404 | 9479040 | 97.69 | 3.38 | 75.58 |
| H45-13 | 1044593400 | 1029758700 | 6865058 | 6706200 | 97.69 | 2.64 | 71.77 |
| L109-54 | 1579314900 | 1544558400 | 10060761 | 10297056 | 97.71 | 3.39 | 79.4 |
| H26-22 | 1404659700 | 1322167500 | 8814450 | 8614499 | 97.73 | 3.12 | 73.65 |
| H8-3 | 1076313900 | 1062718500 | 7084790 | 6925267 | 97.75 | 2.63 | 74.36 |
| H47-2 | 1307631900 | 1284100800 | 8560672 | 8368676 | 97.76 | 3.04 | 73.31 |
| H65-12 | 1265721600 | 1244945100 | 8299634 | 8117771 | 97.81 | 2.97 | 75.87 |
| H7-7 | 1512255900 | 1484223300 | 9894822 | 9678020 | 97.81 | 3.28 | 80.28 |
| H64-1 | 1576197300 | 1548583200 | 10323888 | 10101810 | 97.85 | 3.43 | 80.86 |
| L109-56 | 1636171200 | 1604053200 | 10465921 | 10693688 | 97.87 | 3.55 | 79.22 |
| **Total/Average*** | 662942536500 | 646773351000 | 4280884772 | NA | 95.44* | 2.98* | 74.22* |

**Table S4. The genotype counts of the GWAS-identified candidate SNPs in DIE and SUR group**

| **#CHR** | **Pos** | **Ref** | **Alt** | **DIE group** | | | | | | **SUR group** | | | | | |
| --- | --- | --- | --- | --- | --- | --- | --- | --- | --- | --- | --- | --- | --- | --- | --- |
|  |  |  |  | **0/0 num** | **0/1 num** | **1/1 num** | **0/0 ratio** | **0/1 ratio** | **1/1 ratio** | **0/0 num** | **0/1 num** | **1/1 num** | **0/0 ratio** | **0/1 ratio** | **1/1 ratio** |
| chr2 | 923324 | G | T | 76 | 30 | 1 | 0.710 | 0.280 | 0.009 | 324 | 26 | 2 | 0.920 | 0.074 | 0.006 |
| chr5 | 10827794 | T | G | 86 | 19 | 0 | 0.819 | 0.181 | 0.000 | 351 | 9 | 0 | 0.975 | 0.025 | 0.000 |
| chr5 | 11021059 | C | T | 95 | 13 | 0 | 0.880 | 0.120 | 0.000 | 353 | 3 | 0 | 0.992 | 0.008 | 0.000 |
| chr5 | 11060924 | C | T | 87 | 19 | 0 | 0.821 | 0.179 | 0.000 | 352 | 11 | 0 | 0.970 | 0.030 | 0.000 |
| chr5 | 11704307 | C | T | 83 | 20 | 2 | 0.790 | 0.190 | 0.019 | 352 | 18 | 0 | 0.951 | 0.049 | 0.000 |
| chr5 | 12003828 | G | T | 89 | 19 | 0 | 0.824 | 0.176 | 0.000 | 338 | 9 | 0 | 0.974 | 0.026 | 0.000 |
| chr5 | 14549123 | C | T | 94 | 13 | 0 | 0.879 | 0.121 | 0.000 | 356 | 3 | 0 | 0.992 | 0.008 | 0.000 |
| chr5 | 15676676 | C | G | 95 | 11 | 0 | 0.896 | 0.104 | 0.000 | 355 | 1 | 0 | 0.997 | 0.003 | 0.000 |
| chr5 | 15846474 | C | T | 91 | 21 | 1 | 0.805 | 0.186 | 0.009 | 348 | 15 | 0 | 0.959 | 0.041 | 0.000 |
| chr5 | 15869062 | G | A | 93 | 20 | 0 | 0.823 | 0.177 | 0.000 | 351 | 11 | 0 | 0.970 | 0.030 | 0.000 |
| chr5 | 16002435 | G | C | 85 | 20 | 0 | 0.810 | 0.190 | 0.000 | 351 | 11 | 0 | 0.970 | 0.030 | 0.000 |
| chr5 | 4427074 | T | C | 91 | 19 | 0 | 0.827 | 0.173 | 0.000 | 357 | 9 | 0 | 0.975 | 0.025 | 0.000 |
| chr5 | 4477882 | C | T | 71 | 33 | 0 | 0.683 | 0.317 | 0.000 | 327 | 32 | 1 | 0.908 | 0.089 | 0.003 |
| chr5 | 5026823 | G | T | 87 | 21 | 0 | 0.806 | 0.194 | 0.000 | 346 | 12 | 0 | 0.966 | 0.034 | 0.000 |
| chr5 | 5066899 | G | T | 87 | 21 | 1 | 0.798 | 0.193 | 0.009 | 340 | 12 | 0 | 0.966 | 0.034 | 0.000 |
| chr5 | 5095129 | A | T | 88 | 20 | 1 | 0.807 | 0.183 | 0.009 | 342 | 11 | 0 | 0.969 | 0.031 | 0.000 |
| chr5 | 5160729 | C | T | 85 | 18 | 1 | 0.817 | 0.173 | 0.010 | 350 | 13 | 0 | 0.964 | 0.036 | 0.000 |
| chr5 | 5256553 | C | T | 83 | 24 | 1 | 0.769 | 0.222 | 0.009 | 343 | 19 | 0 | 0.948 | 0.052 | 0.000 |
| chr5 | 5932644 | C | T | 93 | 12 | 0 | 0.886 | 0.114 | 0.000 | 358 | 3 | 0 | 0.992 | 0.008 | 0.000 |
| chr5 | 7190601 | C | T | 91 | 13 | 0 | 0.875 | 0.125 | 0.000 | 356 | 4 | 0 | 0.989 | 0.011 | 0.000 |
| chr5 | 7976244 | A | G | 90 | 13 | 0 | 0.874 | 0.126 | 0.000 | 361 | 4 | 0 | 0.989 | 0.011 | 0.000 |
| chr5 | 8066080 | C | T | 79 | 26 | 0 | 0.752 | 0.248 | 0.000 | 338 | 22 | 0 | 0.939 | 0.061 | 0.000 |
| chr5 | 8456346 | C | A | 97 | 11 | 0 | 0.898 | 0.102 | 0.000 | 359 | 1 | 0 | 0.997 | 0.003 | 0.000 |
| chr7 | 2965356 | G | A | 93 | 18 | 0 | 0.838 | 0.162 | 0.000 | 350 | 9 | 0 | 0.975 | 0.025 | 0.000 |
| chr7 | 3016982 | T | C | 89 | 18 | 0 | 0.832 | 0.168 | 0.000 | 342 | 9 | 0 | 0.974 | 0.026 | 0.000 |
| chr12 | 13536810 | G | A | 97 | 10 | 0 | 0.907 | 0.093 | 0.000 | 362 | 1 | 0 | 0.997 | 0.003 | 0.000 |
| chr12 | 15289909 | C | G | 87 | 22 | 0 | 0.798 | 0.202 | 0.000 | 335 | 13 | 0 | 0.963 | 0.037 | 0.000 |
| chr12 | 7906314 | G | A | 100 | 10 | 0 | 0.909 | 0.091 | 0.000 | 360 | 1 | 0 | 0.997 | 0.003 | 0.000 |
| chr14 | 21529897 | C | T | 84 | 25 | 2 | 0.757 | 0.225 | 0.018 | 329 | 22 | 1 | 0.935 | 0.063 | 0.003 |
| chr14 | 22391750 | A | T | 73 | 34 | 1 | 0.676 | 0.315 | 0.009 | 312 | 37 | 0 | 0.894 | 0.106 | 0.000 |
| chr14 | 24494340 | C | G | 84 | 22 | 0 | 0.792 | 0.208 | 0.000 | 345 | 14 | 0 | 0.961 | 0.039 | 0.000 |
| chr15 | 2694305 | T | C | 93 | 14 | 0 | 0.869 | 0.131 | 0.000 | 348 | 4 | 0 | 0.989 | 0.011 | 0.000 |
| chr17 | 13011075 | C | A | 88 | 17 | 0 | 0.838 | 0.162 | 0.000 | 367 | 6 | 0 | 0.984 | 0.016 | 0.000 |

**Table S5. Genes in the candidate regions identified by Fst and nucleotide diversity analyses**

| **CHR** | **GeneStart** | **GeneEnd** | **GeneID** | **Annotation** | **Gene length** |
| --- | --- | --- | --- | --- | --- |
| chr10 | 1203278 | 1240889 | Cse_R002423 | Inositol 1,4,5-trisphosphate receptor type 3 | 37612 |
| chr10 | 1249889 | 1251763 | Cse_R002503 | Deoxyribonuclease gamma | 1875 |
| chr10 | 1253650 | 1262285 | Cse_R002866 | Minor histocompatibility antigen H13 | 8636 |
| chr10 | 8640452 | 8663572 | Cse_R002928 | E3 ubiquitin-protein ligase MIB2 | 23121 |
| chr10 | 8676901 | 8678731 | Cse_R002172 | UbiA prenyltransferase domain-containing protein 1 | 1831 |
| chr10 | 8683702 | 8688357 | Cse_R002322 | Matrix metalloproteinase-23 | 4656 |
| chr10 | 8691188 | 8701694 | Cse_R002849 | Cyclin-dependent kinase 11 | 10507 |
| chr11 | 2945034 | 2954074 | Cse_R003337 | AMP deaminase 2 | 9041 |
| chr11 | 2958120 | 2962261 | Cse_R003360 | Epidermal growth factor receptor kinase substrate 8-like protein 3 | 4142 |
| chr11 | 2964690 | 2967571 | Cse_R003185 | Interferon regulatory factor 6 | 2882 |
| chr11 | 2971205 | 2974709 | Cse_R003293 | Guanylyl cyclase-activating protein 1 | 3505 |
| chr11 | 3631647 | 3639533 | Cse_R003104 | GATA-binding factor 2 | 7887 |
| chr11 | 6255782 | 6273831 | Cse_R003522 | Tensin-like C1 domain-containing phosphatase | 18050 |
| chr11 | 6288437 | 6296820 | Cse_R003239 | Vitamin D3 receptor B | 8384 |
| chr11 | 10406467 | 10431274 | Cse_R003297 | Contactin-4 | 24808 |
| chr11 | 10504875 | 10589996 | Cse_R003573 | Contactin-4 | 85122 |
| chr11 | 10592373 | 10595634 | Cse_R019578 | PRA1 family protein 3 | 3262 |
| chr11 | 10596044 | 10600653 | Cse_R019582 | CCA tRNA nucleotidyltransferase 1, mitochondrial | 4610 |
| chr11 | 11126197 | 11150315 | Cse_R019106 | FERM domain-containing protein 4B | 24119 |
| chr11 | 11161808 | 11163072 | Cse_R003238 | Contactin-4 | 1265 |
| chr11 | 11170296 | 11195025 | Cse_R003388 | Band 4.1-like protein 1 | 24730 |
| chr11 | 11235325 | 11239334 | Cse_R003714 | Bactericidal permeability-increasing protein | 4010 |
| chr11 | 11239896 | 11245282 | Cse_R003656 | CDK5 regulatory subunit-associated protein 1 | 5387 |
| chr11 | 11246844 | 11250289 | Cse_R003157 | Somatoliberin | 3446 |
| chr11 | 11254063 | 11259714 | Cse_R003648 | Dolichyl-diphosphooligosaccharide--protein glycosyltransferase subunit 2 | 5652 |
| chr11 | 11261673 | 11267595 | Cse_R003218 | Myb-related protein B | 5923 |
| chr11 | 11268754 | 11269218 | Cse_R003836 | -- | 465 |
| chr11 | 11269607 | 11273130 | Cse_R003616 | Intraflagellar transport protein 52 homolog | 3524 |
| chr11 | 11274987 | 11277295 | Cse_R003108 | Acyl-coenzyme A thioesterase 8 | 2309 |
| chr11 | 11288814 | 11300423 | Cse_R003870 | Phosphatase and actin regulator 3 | 11610 |
| chr11 | 11307991 | 11314714 | Cse_R003219 | Solute carrier family 41 member 3 | 6724 |
| chr11 | 11318902 | 11319747 | Cse_R003673 | Carbohydrate sulfotransferase 11 | 846 |
| chr11 | 11331665 | 11342092 | Cse_R003758 | Thioredoxin reductase 3 (Fragment) | 10428 |
| chr11 | 11344388 | 11345571 | Cse_R003677 | Axonemal dynein light intermediate polypeptide 1 | 1184 |
| chr11 | 11347221 | 11347880 | Cse_R003244 | Charged multivesicular body protein 3 | 660 |
| chr11 | 11355240 | 11357974 | Cse_R003667 | Coiled-coil domain-containing protein 120 | 2735 |
| chr11 | 11372249 | 11379311 | Cse_R003042 | Methyl-CpG-binding domain protein 1 | 7063 |
| chr11 | 11382108 | 11386245 | Cse_R003879 | CpG-binding protein | 4138 |
| chr11 | 11398982 | 11407879 | Cse_R003071 | Nuclear receptor coactivator 3 | 8898 |
| chr11 | 11413598 | 11414176 | Cse_R003650 | DNA-binding protein inhibitor ID-1 | 579 |
| chr11 | 13856587 | 13874405 | Cse_R003693 | Lysine-specific demethylase PHF2 | 17819 |
| chr11 | 13905972 | 13908786 | Cse_R003796 | Homeobox protein BarH-like 1 | 2815 |
| chr11 | 13912542 | 13915482 | Cse_R003167 | Carbohydrate sulfotransferase 15 | 2941 |
| chr12 | 4391928 | 4400342 | Cse_R004026 | Pleckstrin homology domain-containing family A member 1 | 8415 |
| chr12 | 4408370 | 4430998 | Cse_R003902 | Serine protease HTRA1A | 22629 |
| chr12 | 4435099 | 4449450 | Cse_R004285 | Wings apart-like protein homolog | 14352 |
| chr12 | 7025163 | 7061451 | Cse_R019052 | KH domain-containing, RNA-binding, signal transduction-associated protein 2 | 36289 |
| chr12 | 7073949 | 7076958 | Cse_R019060 | L-asparaginase | 3010 |
| chr12 | 7078557 | 7085222 | Cse_R019053 | PHD finger protein 3 | 6666 |
| chr12 | 7086974 | 7212987 | Cse_R019071 | Protein eyes shut homolog | 126014 |
| chr12 | 7823279 | 8022282 | Cse_R019064 | Neurexin-1a-alpha | 199004 |
| chr12 | 8956342 | 8969857 | Cse_R003896 | Serine palmitoyltransferase 3 | 13516 |
| chr12 | 9977025 | 9985768 | Cse_R004331 | Kv channel-interacting protein 2 | 8744 |
| chr12 | 9988919 | 9990785 | Cse_R004316 | Cytochrome c oxidase assembly protein COX15 homolog | 1867 |
| chr12 | 9992394 | 9994740 | Cse_R004082 | Copper homeostasis protein cutC homolog | 2347 |
| chr12 | 9999266 | 10012409 | Cse_R004324 | Canalicular multispecific organic anion transporter 1 | 13144 |
| chr12 | 10017884 | 10020651 | Cse_R004059 | Rho GTPase-activating protein 19 | 2768 |
| chr12 | 10028917 | 10112397 | Cse_R004321 | Slit homolog 1 protein | 83481 |
| chr12 | 10892381 | 10905454 | Cse_R021555 | Protein FAM204A | 13074 |
| chr12 | 11168162 | 11204366 | Cse_R018773 | DNA nucleotidylexotransferase | 36205 |
| chr12 | 11419071 | 11422720 | Cse_R018793 | Cytochrome P450 1B1 | 3650 |
| chr12 | 11443699 | 11444860 | Cse_R018781 | N-acylneuraminate-9-phosphatase | 1162 |
| chr12 | 11445325 | 11451290 | Cse_R018775 | DNA-directed RNA polymerase I subunit RPA2 | 5966 |
| chr12 | 11454436 | 11457258 | Cse_R018774 | Tubulin--tyrosine ligase | 2823 |
| chr12 | 11459682 | 11461645 | Cse_R018776 | Fidgetin-like protein 1 | 1964 |
| chr12 | 11467714 | 11481289 | Cse_R018777 | DNA-binding protein Ikaros | 13576 |
| chr12 | 11534779 | 11539415 | Cse_R018778 | Brorin | 4637 |
| chr12 | 11561868 | 11570429 | Cse_R018802 | Poly(rC)-binding protein 3 | 8562 |
| chr12 | 11578045 | 11585950 | Cse_R018800 | Molybdenum cofactor sulfurase | 7906 |
| chr12 | 11592398 | 11594349 | Cse_R018780 | Monoacylglycerol lipase ABHD12 | 1952 |
| chr12 | 11598826 | 11605460 | Cse_R018788 | Transmembrane 9 superfamily member 3 | 6635 |
| chr12 | 11965142 | 12062794 | Cse_R021777 | Type I inositol-1,4,5-trisphosphate 5-phosphatase | 97653 |
| chr12 | 12385189 | 12400232 | Cse_R018488 | Calpain-2 catalytic subunit | 15044 |
| chr12 | 12404881 | 12410442 | Cse_R018497 | Calpain-1 catalytic subunit | 5562 |
| chr12 | 12411803 | 12412612 | Cse_R018496 | 39S ribosomal protein L14, mitochondrial | 810 |
| chr12 | 12418534 | 12428815 | Cse_R018489 | Transmembrane protein 63B | 10282 |
| chr12 | 12431588 | 12436527 | Cse_R018498 | Zinc transporter ZIP9 | 4940 |
| chr12 | 12438540 | 12440222 | Cse_R018494 | Uncharacterized protein C1orf65 | 1683 |
| chr12 | 13080361 | 13085661 | Cse_R021896 | Forkhead box protein N2 | 5301 |
| chr12 | 13090942 | 13098442 | Cse_R021883 | Protein phosphatase 1 regulatory subunit 21 | 7501 |
| chr12 | 13105038 | 13107285 | Cse_R021894 | Stonin-1 | 2248 |
| chr12 | 13111725 | 13115288 | Cse_R021483 | Hyaluronan-binding protein 2 | 3564 |
| chr12 | 13116519 | 13118798 | Cse_R021482 | Nuclear factor 7, ovary | 2280 |
| chr12 | 13120109 | 13124543 | Cse_R021481 | Delta-1-pyrroline-5-carboxylate synthase | 4435 |
| chr12 | 13125141 | 13168518 | Cse_R021484 | Arginyl-tRNA--protein transferase 1 | 43378 |
| chr12 | 13197859 | 13200062 | Cse_R021486 | -- | 2204 |
| chr12 | 13200187 | 13211170 | Cse_R021485 | Fibroblast growth factor receptor 2 | 10984 |
| chr12 | 13222133 | 13233603 | Cse_R004049 | -- | 11471 |
| chr12 | 13241137 | 13243331 | Cse_R004412 | McKusick-Kaufman/Bardet-Biedl syndromes putative chaperonin | 2195 |
| chr12 | 13248022 | 13251401 | Cse_R004280 | Protein MEMO1 | 3380 |
| chr12 | 13253395 | 13256824 | Cse_R004024 | spastin | 3430 |
| chr12 | 13270853 | 13276940 | Cse_R004305 | Opsin-5 | 6088 |
| chr12 | 13283389 | 13300317 | Cse_R003892 | Vitrin | 16929 |
| chr12 | 13303462 | 13328111 | Cse_R004290 | Striatin | 24650 |
| chr12 | 13329698 | 13347901 | Cse_R003938 | HEAT repeat-containing protein 5B | 18204 |
| chr12 | 13348630 | 13349059 | Cse_R004003 | Coiled-coil domain-containing protein 75 | 430 |
| chr12 | 13348672 | 13350672 | Cse_R004264 | Coiled-coil domain-containing protein 75 | 2001 |
| chr12 | 13352470 | 13363911 | Cse_R004417 | Interferon-induced, double-stranded RNA-activated protein kinase | 11442 |
| chr12 | 13563771 | 13600572 | Cse_R004338 | Lysosomal-trafficking regulator | 36802 |
| chr12 | 13617001 | 13632326 | Cse_R004279 | Nidogen-1 | 15326 |
| chr12 | 13635226 | 13639249 | Cse_R003980 | Integral membrane protein GPR137B | 4024 |
| chr12 | 14173444 | 14194256 | Cse_R004074 | Inactive tyrosine-protein kinase 7 | 20813 |
| chr12 | 14198682 | 14217971 | Cse_R004345 | Serum response factor | 19290 |
| chr12 | 14223264 | 14230307 | Cse_R004414 | Protein FAM160B1 | 7044 |
| chr12 | 14236853 | 14259146 | Cse_R004117 | Actin-binding LIM protein 1 | 22294 |
| chr12 | 14267927 | 14269157 | Cse_R004177 | Zinc-binding protein A33 | 1231 |
| chr12 | 14278485 | 14314126 | Cse_R003934 | spectrin beta chain, brain 1 | 35642 |
| chr12 | 15268542 | 15289434 | Cse_R004079 | WD repeat-containing protein 96 | 20893 |
| chr12 | 15289567 | 15290322 | Cse_R004022 | Swi5-dependent recombination DNA repair protein 1 homolog | 756 |
| chr12 | 15305384 | 15327097 | Cse_R004332 | Collagen alpha-1(XVII) chain | 21714 |
| chr12 | 15337082 | 15343726 | Cse_R004046 | Phosphatidylinositol-3,4,5-trisphosphate 3-phosphatase and dual-specificity protein phosphatase PTEN | 6645 |
| chr12 | 16036799 | 16120963 | Cse_R004436 | Baculoviral IAP repeat-containing protein 6 | 84165 |
| chr15 | 1235774 | 1260618 | Cse_R006425 | Dystrophin-related protein 2 | 24845 |
| chr15 | 4523027 | 4553174 | Cse_R017374 | Ankyrin repeat domain-containing protein 50 | 30148 |
| chr15 | 10621795 | 10628530 | Cse_R006291 | E3 ubiquitin-protein ligase BRE1A | 6736 |
| chr15 | 10631597 | 10633502 | Cse_R006501 | Fructose-bisphosphate aldolase B | 1906 |
| chr15 | 10634775 | 10636187 | Cse_R006136 | -- | 1413 |
| chr15 | 10637556 | 10640624 | Cse_R006814 | Transmembrane and coiled-coil domain-containing protein 6 | 3069 |
| chr15 | 10651159 | 10653094 | Cse_R006249 | Arylsulfatase I | 1936 |
| chr16 | 5192677 | 5241911 | Cse_R017393 | Sodium leak channel non-selective protein | 49235 |
| chr16 | 5249015 | 5274191 | Cse_R017377 | Integrin beta-like protein 1 | 25177 |
| chr16 | 7829746 | 7955187 | Cse_R018435 | Dachshund homolog 1 | 125442 |
| chr16 | 16876144 | 16926944 | Cse_R007288 | Serum deprivation-response protein | 50801 |
| chr16 | 16934626 | 17017269 | Cse_R007453 | Tomoregulin-2 | 82644 |
| chr16 | 17864314 | 17878235 | Cse_R007480 | Double-stranded RNA-specific editase 1 | 13922 |
| chr16 | 17886391 | 17888749 | Cse_R007260 | Anterior gradient protein 3 homolog | 2359 |
| chr16 | 17890442 | 17904204 | Cse_R007483 | TRAF3-interacting protein 1 | 13763 |
| chr16 | 17908914 | 17911907 | Cse_R007424 | Ankyrin repeat and SOCS box protein 1 | 2994 |
| chr16 | 17915976 | 17916401 | Cse_R007048 | Protein FEV | 426 |
| chr16 | 17922644 | 17924773 | Cse_R007509 | Beta-crystallin A2 | 2130 |
| chr16 | 17934746 | 17938179 | Cse_R007530 | Uridine 5'-monophosphate synthase | 3434 |
| chr16 | 17940784 | 17965178 | Cse_R007091 | Fibronectin | 24395 |
| chr16 | 17984515 | 17989940 | Cse_R007518 | Melanoregulin | 5426 |
| chr16 | 18035051 | 18104864 | Cse_R007235 | Partitioning defective 3 homolog B | 69814 |
| chr16 | 18117540 | 18142122 | Cse_R006947 | Partitioning defective 3 homolog B | 24583 |
| chr16 | 18181071 | 18239568 | Cse_R007216 | Neuropilin-2 | 58498 |
| chr17 | 3137493 | 3144530 | Cse_R008436 | Mitogen-activated protein kinase kinase kinase 3 | 7038 |
| chr17 | 3149296 | 3153216 | Cse_R007866 | Glial fibrillary acidic protein | 3921 |
| chr17 | 3164323 | 3180152 | Cse_R007822 | Potassium voltage-gated channel subfamily H member 7 | 15830 |
| chr17 | 3939422 | 3991741 | Cse_R007931 | Voltage-dependent L-type calcium channel subunit beta-1 | 52320 |
| chr17 | 3998259 | 4000117 | Cse_R007696 | 60S ribosomal protein L19 | 1859 |
| chr17 | 5031232 | 5094343 | Cse_R008021 | BAH and coiled-coil domain-containing protein 1 | 63112 |
| chr17 | 5866308 | 5910290 | Cse_R007952 | Latrophilin-1 | 43983 |
| chr17 | 7557827 | 7565003 | Cse_R007849 | DNA polymerase delta catalytic subunit | 7177 |
| chr17 | 7570787 | 7576578 | Cse_R008149 | Glycogen [starch] synthase, muscle | 5792 |
| chr17 | 7577788 | 7578219 | Cse_R008041 | Putative L-aspartate dehydrogenase | 432 |
| chr17 | 7578971 | 7581028 | Cse_R008192 | Putative L-aspartate dehydrogenase | 2058 |
| chr17 | 7582037 | 7583804 | Cse_R008157 | Lysoplasmalogenase | 1768 |
| chr17 | 7584473 | 7586216 | Cse_R007592 | Hsp70-binding protein 1 | 1744 |
| chr17 | 7588610 | 7591015 | Cse_R007832 | Elongation factor Tu, mitochondrial | 2406 |
| chr17 | 7595578 | 7596813 | Cse_R007881 | ATP-sensitive inward rectifier potassium channel 12 | 1236 |
| chr17 | 7607503 | 7607881 | Cse_R007936 | Protein GTLF3B | 379 |
| chr17 | 7611626 | 7612141 | Cse_R007747 | Transmembrane protein 11, mitochondrial | 516 |
| chr17 | 7616128 | 7617993 | Cse_R007633 | Dehydrogenase/reductase SDR family member 7B | 1866 |
| chr17 | 8377577 | 8379370 | Cse_R007785 | Transcription factor Sox-8 | 1794 |
| chr17 | 8410455 | 8411368 | Cse_R007741 | Dexamethasone-induced Ras-related protein 1 | 914 |
| chr17 | 8412433 | 8412905 | Cse_R008440 | Mediator of RNA polymerase II transcription subunit 9 | 473 |
| chr17 | 8417028 | 8434778 | Cse_R008296 | Ubiquitin carboxyl-terminal hydrolase 22 | 17751 |
| chr17 | 10702681 | 10705043 | Cse_R008042 | THO complex subunit 4-A | 2363 |
| chr17 | 10706633 | 10707317 | Cse_R007927 | Protein FAM195B | 685 |
| chr17 | 10709993 | 10711249 | Cse_R007670 | Protein phosphatase 1 regulatory subunit 27 | 1257 |
| chr17 | 10720655 | 10725437 | Cse_R008016 | Platelet endothelial cell adhesion molecule | 4783 |
| chr17 | 11299618 | 11321932 | Cse_R008182 | Lethal(2) giant larvae protein homolog 1 | 22315 |
| chr17 | 11323389 | 11350036 | Cse_R008275 | Unconventional myosin-XV | 26648 |
| chr17 | 11357513 | 11360095 | Cse_R008330 | Developmentally-regulated GTP-binding protein 2 | 2583 |
| chr17 | 11360608 | 11361476 | Cse_R007582 | FAD-linked sulfhydryl oxidase ALR | 869 |
| chr17 | 11362174 | 11365895 | Cse_R008053 | Synaptogyrin-3 | 3722 |
| chr17 | 11370590 | 11375444 | Cse_R008332 | Zinc finger protein 598 | 4855 |
| chr17 | 11376151 | 11378138 | Cse_R008158 | Peroxisomal membrane protein PMP34 | 1988 |
| chr17 | 11379333 | 11381537 | Cse_R008242 | RNA pseudouridylate synthase domain-containing protein 1 | 2205 |
| chr17 | 11388045 | 11404855 | Cse_R007567 | Rho GDP-dissociation inhibitor 1 | 16811 |
| chr17 | 11411589 | 11421792 | Cse_R007948 | H(+)/Cl(-) exchange transporter 7 | 10204 |
| chr17 | 11422781 | 11423428 | Cse_R008203 | Pentraxin-4 | 648 |
| chr17 | 11427884 | 11430143 | Cse_R007819 | Solute carrier family 2, facilitated glucose transporter member 6 | 2260 |
| chr17 | 11437861 | 11438494 | Cse_R007891 | Lipopolysaccharide-induced tumor necrosis factor-alpha factor homolog | 634 |
| chr17 | 11441888 | 11457091 | Cse_R007808 | Ubiquitin carboxyl-terminal hydrolase 7 | 15204 |
| chr17 | 11460251 | 11463723 | Cse_R007815 | UPF0472 protein C16orf72 homolog | 3473 |
| chr17 | 11468357 | 11470171 | Cse_R008159 | Dynactin subunit 5 | 1815 |
| chr17 | 11470724 | 11472306 | Cse_R007846 | Acyl carrier protein, mitochondrial | 1583 |
| chr17 | 11480316 | 11490603 | Cse_R008205 | Trinucleotide repeat-containing gene 6A protein | 10288 |
| chr17 | 12615343 | 12620285 | Cse_R007693 | TRIO and F-actin-binding protein | 4943 |
| chr17 | 12621790 | 12623082 | Cse_R007983 | NADH dehydrogenase [ubiquinone] 1 alpha subcomplex subunit 6 | 1293 |
| chr17 | 12624211 | 12624653 | Cse_R008082 | UPF0466 protein C22orf32, mitochondrial | 443 |
| chr17 | 12627019 | 12627461 | Cse_R008373 | UPF0466 protein C22orf32, mitochondrial | 443 |
| chr17 | 12628662 | 12633979 | Cse_R007673 | Uncharacterized protein KIAA1467 homolog | 5318 |
| chr17 | 12636108 | 12638691 | Cse_R008356 | Polymerase delta-interacting protein 3 | 2584 |
| chr17 | 12639874 | 12641414 | Cse_R008223 | Ribosomal RNA-processing protein 7 homolog A | 1541 |
| chr17 | 12642143 | 12645050 | Cse_R008238 | Zona pellucida sperm-binding protein 4 | 2908 |
| chr17 | 12646007 | 12648975 | Cse_R007572 | WW domain-binding protein 2 | 2969 |
| chr17 | 12652167 | 12656929 | Cse_R008104 | Heme-binding protein 1 | 4763 |
| chr17 | 12658266 | 12659506 | Cse_R007720 | UPF0562 protein C7orf55 homolog | 1241 |
| chr17 | 12690685 | 12714693 | Cse_R007818 | Glutamate [NMDA] receptor subunit epsilon-2 | 24009 |
| chr17 | 12723826 | 12749493 | Cse_R008113 | Glutamate [NMDA] receptor subunit epsilon-2 | 25668 |
| chr17 | 12773854 | 12780844 | Cse_R008045 | Transcription factor 20 | 6991 |
| chr17 | 12785671 | 12788123 | Cse_R008420 | Germ cell-specific gene 1-like protein | 2453 |
| chr17 | 12810761 | 12813662 | Cse_R007911 | Epithelial membrane protein 2 | 2902 |
| chr17 | 12824940 | 12826944 | Cse_R007941 | -- | 2005 |
| chr17 | 12832954 | 12835337 | Cse_R008106 | CD9 antigen | 2384 |
| chr17 | 12853931 | 12880839 | Cse_R008266 | RNA binding protein fox-1 homolog 2 | 26909 |
| chr17 | 12888630 | 12889598 | Cse_R008212 | G-protein coupled receptor family C group 5 member C | 969 |
| chr17 | 12893016 | 12893543 | Cse_R007917 | Retinal cone rhodopsin-sensitive cGMP 3',5'-cyclic phosphodiesterase subunit gamma | 528 |
| chr17 | 12919281 | 12921747 | Cse_R007649 | Matrix Gla protein | 2467 |
| chr17 | 12926724 | 12927827 | Cse_R008189 | Pleckstrin homology domain-containing family F member 1 | 1104 |
| chr17 | 12930596 | 12931652 | Cse_R008066 | Serotonin N-acetyltransferase | 1057 |
| chr17 | 12934465 | 12937780 | Cse_R008012 | WW domain-binding protein 11 | 3316 |
| chr17 | 12939093 | 12942057 | Cse_R008328 | Probable ATP-dependent RNA helicase DDX47 | 2965 |
| chr17 | 12944726 | 12945091 | Cse_R007734 | Myosin-10 | 366 |
| chr17 | 12961781 | 12980345 | Cse_R008318 | Myosin-10 | 18565 |
| chr17 | 12984955 | 12991742 | Cse_R008226 | Caskin-2 | 6788 |
| chr17 | 12994163 | 12996177 | Cse_R008084 | THO complex subunit 6 homolog | 2015 |
| chr17 | 12996646 | 12998847 | Cse_R007898 | Mediator of RNA polymerase II transcription subunit 15 | 2202 |
| chr17 | 12998794 | 13004280 | Cse_R008461 | Mediator of RNA polymerase II transcription subunit 15 | 5487 |
| chr17 | 13007294 | 13010930 | Cse_R008453 | F-box/LRR-repeat protein 19 | 3637 |
| chr17 | 13012576 | 13016010 | Cse_R008168 | NEDD8-conjugating enzyme Ubc12 | 3435 |
| chr17 | 13017961 | 13022325 | Cse_R008006 | E3 ubiquitin/ISG15 ligase TRIM25 | 4365 |
| chr17 | 13026716 | 13032059 | Cse_R007599 | Rho GTPase-activating protein 27 | 5344 |
| chr17 | 13034555 | 13041184 | Cse_R007739 | Importin subunit beta-1 | 6630 |
| chr17 | 14540181 | 14544618 | Cse_R008001 | Zinc finger protein 653 | 4438 |
| chr17 | 14545192 | 14547166 | Cse_R008245 | RPA-interacting protein A | 1975 |
| chr17 | 14547855 | 14551357 | Cse_R008355 | 26S proteasome non-ATPase regulatory subunit 11 | 3503 |
| chr17 | 14554329 | 14560613 | Cse_R007634 | Homeobox protein Dlx3b | 6285 |
| chr17 | 14570072 | 14576477 | Cse_R008069 | Homeobox protein Dlx4a | 6406 |
| chr17 | 14613409 | 14615486 | Cse_R007698 | N-acetylglutamate synthase, mitochondrial | 2078 |
| chr17 | 14616824 | 14617635 | Cse_R008062 | Coiled-coil domain-containing protein 56 | 812 |
| chr17 | 14647950 | 14650098 | Cse_R007671 | Collagen alpha-1(I) chain | 2149 |
| chr17 | 14647950 | 14652831 | Cse_R008146 | Collagen alpha-1(I) chain | 4882 |
| chr17 | 14653115 | 14656054 | Cse_R007624 | -- | 2940 |
| chr17 | 14656428 | 14657000 | Cse_R007880 | -- | 573 |
| chr17 | 14658302 | 14710901 | Cse_R007563 | Macrophage receptor MARCO | 52600 |
| chr17 | 14728131 | 14745024 | Cse_R007653 | Protein TANC2 | 16894 |
| chr17 | 14748530 | 14753698 | Cse_R008133 | Cytochrome b561 | 5169 |
| chr17 | 14779875 | 14794654 | Cse_R008072 | Histone deacetylase 5 | 14780 |
| chr17 | 14799146 | 14800457 | Cse_R007996 | Peroxisome assembly protein 12 | 1312 |
| chr17 | 14801841 | 14805722 | Cse_R008099 | TLR4 interactor with leucine rich repeats | 3882 |
| chr17 | 14807707 | 14810657 | Cse_R008210 | T-cell receptor beta chain T17T-22 | 2951 |
| chr17 | 14812870 | 14817118 | Cse_R008117 | MAGUK p55 subfamily member 2 | 4249 |
| chr17 | 14819098 | 14822858 | Cse_R008425 | Putative all-trans-retinol 13,14-reductase | 3761 |
| chr17 | 15756204 | 15757684 | Cse_R008052 | Transcription factor jun-B | 1481 |
| chr17 | 15761580 | 15762451 | Cse_R007945 | Uncharacterized protein C19orf43 | 872 |
| chr17 | 15764138 | 15770722 | Cse_R008173 | Ubinuclein-2 | 6585 |
| chr17 | 15771935 | 15773755 | Cse_R008088 | 40S ribosomal protein SA | 1821 |
| chr17 | 15775660 | 15777045 | Cse_R008156 | Solute carrier family 25 member 38-A | 1386 |
| chr17 | 15780229 | 15826903 | Cse_R008044 | Myosin-9 | 46675 |
| chr17 | 15828908 | 15833109 | Cse_R008193 | Leucine-rich repeat-containing protein 8D | 4202 |
| chr17 | 15842008 | 15842310 | Cse_R008281 | Zinc finger protein Gfi-1 | 303 |
| chr17 | 15844200 | 15850929 | Cse_R008325 | Transforming growth factor beta receptor type 3 | 6730 |
| chr17 | 15852921 | 15854768 | Cse_R007992 | Troponin T, fast skeletal muscle isoforms | 1848 |
| chr17 | 15859938 | 15864058 | Cse_R008197 | PIH1 domain-containing protein 1 | 4121 |
| chr17 | 15877573 | 15888237 | Cse_R008031 | Carnitine O-palmitoyltransferase 1, liver isoform | 10665 |
| chr17 | 15896299 | 15913576 | Cse_R007637 | Serine/threonine-protein kinase BRSK1 | 17278 |
| chr17 | 15930853 | 15931836 | Cse_R008345 | Prolactin | 984 |
| chr17 | 15932783 | 15933960 | Cse_R008137 | Dual specificity protein phosphatase 3 | 1178 |
| chr17 | 15944892 | 15947477 | Cse_R008219 | Thyroid hormone receptor alpha | 2586 |
| chr17 | 15976914 | 15983100 | Cse_R008362 | Vesicle-fusing ATPase | 6187 |
| chr17 | 15992058 | 15995230 | Cse_R007729 | Cyclin-dependent kinase 5 activator 1 | 3173 |
| chr17 | 15997850 | 16000146 | Cse_R007709 | Erythropoietin receptor | 2297 |
| chr17 | 16003364 | 16007190 | Cse_R008443 | Ras-related protein Rab-3D | 3827 |
| chr17 | 16014914 | 16015655 | Cse_R007702 | Transmembrane protein 205 | 742 |
| chr17 | 16018524 | 16018982 | Cse_R008063 | Transcription elongation factor 1 homolog | 459 |
| chr17 | 16021137 | 16022620 | Cse_R008417 | Calponin-1 | 1484 |
| chr17 | 16039596 | 16041079 | Cse_R007857 | Transmembrane protein 56-B | 1484 |
| chr17 | 16098104 | 16107140 | Cse_R007831 | Lipid phosphate phosphatase-related protein type 5 | 9037 |
| chr17 | 16129549 | 16131246 | Cse_R007888 | Protein B602L | 1698 |
| chr17 | 16134266 | 16135225 | Cse_R008377 | Nuclear factor interleukin-3-regulated protein | 960 |
| chr17 | 16143396 | 16145054 | Cse_R007676 | Nuclear factor interleukin-3-regulated protein | 1659 |
| chr17 | 16151984 | 16152508 | Cse_R008283 | Cleavage stimulation factor subunit 2 | 525 |
| chr17 | 16161612 | 16167619 | Cse_R008326 | Ral guanine nucleotide dissociation stimulator-like 1 | 6008 |
| chr17 | 16171930 | 16177413 | Cse_R007937 | Choline transporter-like protein 2 | 5484 |
| chr17 | 16423823 | 16435169 | Cse_R008396 | Neurabin-2 | 11347 |
| chr17 | 16440389 | 16443411 | Cse_R007938 | Phosphoribosyl pyrophosphate synthase-associated protein 2 | 3023 |
| chr18 | 261539 | 269086 | Cse_R008916 | Leucine-rich repeat-containing protein 16A | 7548 |
| chr18 | 274653 | 275633 | Cse_R008944 | -- | 981 |
| chr18 | 276167 | 277531 | Cse_R008696 | Protein N-terminal glutamine amidohydrolase | 1365 |
| chr18 | 278568 | 283068 | Cse_R009030 | Ribosome biogenesis protein bop1 | 4501 |
| chr18 | 284584 | 288452 | Cse_R009051 | Heat shock factor protein 1 | 3869 |
| chr18 | 6506830 | 6523372 | Cse_R008506 | CMP-N-acetylneuraminate-beta-galactosamide-alpha-2,3-sialyltransferase 1 | 16543 |
| chr18 | 6527245 | 6532097 | Cse_R008747 | Poly [ADP-ribose] polymerase 10 | 4853 |
| chr18 | 6533534 | 6542149 | Cse_R008609 | Cleavage and polyadenylation specificity factor subunit 1 | 8616 |
| chr18 | 6542893 | 6545734 | Cse_R008776 | Uncharacterized aarF domain-containing protein kinase 5 | 2842 |
| chr18 | 6547638 | 6558247 | Cse_R008737 | RNA polymerase II subunit A C-terminal domain phosphatase | 10610 |
| chr19 | 9126213 | 9126651 | Cse_R019522 | -- | 439 |
| chr19 | 9130347 | 9132718 | Cse_R019535 | Heat shock protein beta-1 | 2372 |
| chr19 | 9141029 | 9157516 | Cse_R019520 | Serine/arginine repetitive matrix protein 3 | 16488 |
| chr2 | 9655640 | 9688041 | Cse_R021535 | Protein furry homolog-like | 32402 |
| chr2 | 9690277 | 9691932 | Cse_R021538 | Zygote arrest protein 1 | 1656 |
| chr2 | 9692993 | 9695991 | Cse_R021540 | Sodium/bile acid cotransporter 4 | 2999 |
| chr2 | 9704508 | 9706140 | Cse_R021532 | YjeF N-terminal domain-containing protein 3 | 1633 |
| chr2 | 9717169 | 9717481 | Cse_R021539 | Unconventional myosin-Va | 313 |
| chr2 | 9723394 | 9736150 | Cse_R017905 | SLAIN motif-containing protein 2 | 12757 |
| chr2 | 9737110 | 9743156 | Cse_R017920 | Anaphase-promoting complex subunit 4 | 6047 |
| chr2 | 9744556 | 9753031 | Cse_R017909 | Fibrillin-1 | 8476 |
| chr2 | 9758320 | 9765755 | Cse_R017921 | Growth hormone-releasing hormone receptor | 7436 |
| chr2 | 9774095 | 9783978 | Cse_R017904 | FYVE and coiled-coil domain-containing protein 1 | 9884 |
| chr5 | 4099634 | 4103503 | Cse_R012356 | Dihydroorotate dehydrogenase (quinone), mitochondrial | 3870 |
| chr5 | 4110338 | 4111579 | Cse_R012560 | Alpha-1A adrenergic receptor | 1242 |
| chr5 | 4125219 | 4126994 | Cse_R012559 | Haptoglobin | 1776 |
| chr5 | 4131272 | 4136239 | Cse_R012776 | Sulfotransferase family cytosolic 2B member 1 | 4968 |
| chr5 | 4136596 | 4138677 | Cse_R012286 | Dipeptidase 1 | 2082 |
| chr5 | 4139644 | 4141225 | Cse_R012241 | Cleavage and polyadenylation specificity factor subunit 5 | 1582 |
| chr5 | 4143515 | 4147997 | Cse_R012319 | 2-oxoglutarate and iron-dependent oxygenase domain-containing protein 1 | 4483 |
| chr5 | 4151611 | 4154083 | Cse_R012592 | Tumor necrosis factor receptor type 1-associated DEATH domain protein | 2473 |
| chr5 | 4156688 | 4162246 | Cse_R012374 | Guanine nucleotide-binding protein G(o) subunit alpha | 5559 |
| chr5 | 4166140 | 4173241 | Cse_R012638 | Conserved oligomeric Golgi complex subunit 4 | 7102 |
| chr5 | 4177011 | 4201925 | Cse_R012461 | Protein Smaug homolog 1 | 24915 |
| chr5 | 4213460 | 4221049 | Cse_R012418 | GTP cyclohydrolase 1 | 7590 |
| chr5 | 4221826 | 4235157 | Cse_R012625 | WD repeat and HMG-box DNA-binding protein 1 | 13332 |
| chr5 | 4241773 | 4250183 | Cse_R012242 | Disintegrin and metalloproteinase domain-containing protein 10 | 8411 |
| chr5 | 4255356 | 4266051 | Cse_R012544 | Protein FAM63B | 10696 |
| chr5 | 4273026 | 4281447 | Cse_R012694 | SAFB-like transcription modulator | 8422 |
| chr5 | 4284854 | 4297759 | Cse_R012287 | E3 ubiquitin-protein ligase arkadia-C | 12906 |
| chr5 | 4303236 | 4327119 | Cse_R012512 | Unconventional myosin-Ie | 23884 |
| chr5 | 4348816 | 4355389 | Cse_R012631 | Myotubularin-related protein 10 | 6574 |
| chr5 | 4359508 | 4362376 | Cse_R012249 | Ataxin-1-like | 2869 |
| chr5 | 4371138 | 4373551 | Cse_R012304 | Zinc finger protein 821 | 2414 |
| chr5 | 4387543 | 4395898 | Cse_R012323 | AP-1 complex subunit gamma-1 | 8356 |
| chr5 | 4403577 | 4417838 | Cse_R012325 | PH domain leucine-rich repeat-containing protein phosphatase 2 | 14262 |
| chr5 | 4423430 | 4425086 | Cse_R012263 | MARVEL domain-containing protein 3 | 1657 |
| chr5 | 4430531 | 4440230 | Cse_R012550 | Ubiquitin carboxyl-terminal hydrolase 10 | 9700 |
| chr5 | 4448247 | 4457675 | Cse_R012549 | Cysteine-rich secretory protein LCCL domain-containing 2 | 9429 |
| chr5 | 6113065 | 6117955 | Cse_R021965 | Probable methylthioribulose-1-phosphate dehydratase | 4891 |
| chr5 | 7648307 | 7692542 | Cse_R012627 | Glypican-5 | 44236 |
| chr5 | 7991473 | 7997635 | Cse_R012370 | Sodium/hydrogen exchanger 5 | 6163 |
| chr5 | 8000009 | 8017856 | Cse_R012737 | FH1/FH2 domain-containing protein 3 | 17848 |
| chr5 | 8029158 | 8029652 | Cse_R012387 | CCAAT/enhancer-binding protein gamma | 495 |
| chr5 | 8033636 | 8034574 | Cse_R012469 | CCAAT/enhancer-binding protein alpha | 939 |
| chr5 | 8459433 | 8501872 | Cse_R012380 | Protein KIAA1199 | 42440 |
| chr5 | 9616849 | 9739620 | Cse_R019329 | CUGBP Elav-like family member 3-A | 122772 |
| chr5 | 10487922 | 10496040 | Cse_R019779 | CD81 protein | 8119 |
| chr5 | 10510496 | 10515831 | Cse_R019765 | Plakophilin-3 | 5336 |
| chr5 | 10530485 | 10536819 | Cse_R017644 | Iroquois-class homeodomain protein irx-3 | 6335 |
| chr5 | 10579972 | 10672896 | Cse_R017648 | Alpha-ketoglutarate-dependent dioxygenase FTO | 92925 |
| chr5 | 10752850 | 10754642 | Cse_R017650 | AKT-interacting protein | 1793 |
| chr5 | 10763072 | 10763485 | Cse_R017649 | Chromodomain-helicase-DNA-binding protein 9 | 414 |
| chr5 | 10780612 | 10816443 | Cse_R017646 | Chromodomain-helicase-DNA-binding protein 9 | 35832 |
| chr5 | 10822411 | 10823186 | Cse_R017645 | Retinoblastoma-like protein 1 | 776 |
| chr5 | 10955212 | 10963719 | Cse_R017647 | TOX high mobility group box family member 3 | 8508 |
| chr5 | 11133868 | 11143873 | Cse_R017651 | Sal-like protein 1 | 10006 |
| chr5 | 11412017 | 11420325 | Cse_R012300 | Homer protein homolog 2 | 8309 |
| chr5 | 11429890 | 11430514 | Cse_R012659 | WASP homolog-associated protein with actin, membranes and microtubules | 625 |
| chr5 | 11432312 | 11436159 | Cse_R012322 | WASP homolog-associated protein with actin, membranes and microtubules | 3848 |
| chr5 | 11439500 | 11444176 | Cse_R012521 | Fibronectin type III and SPRY domain-containing protein 2 | 4677 |
| chr5 | 11447171 | 11451959 | Cse_R012591 | UPF0469 protein KIAA0907 homolog | 4789 |
| chr5 | 11455673 | 11472171 | Cse_R012265 | High affinity cAMP-specific and IBMX-insensitive 3',5'-cyclic phosphodiesterase 8A | 16499 |
| chr5 | 11750789 | 11754643 | Cse_R012738 | G1/S-specific cyclin-D1 | 3855 |
| chr5 | 12014035 | 12026845 | Cse_R012396 | NAD(P) transhydrogenase, mitochondrial | 12811 |
| chr5 | 12027165 | 12030221 | Cse_R012498 | DTW domain-containing protein 1 | 3057 |
| chr5 | 12038560 | 12041886 | Cse_R012769 | Fibroblast growth factor 7 | 3327 |
| chr5 | 12066004 | 12071143 | Cse_R012601 | COP9 signalosome complex subunit 2 | 5140 |
| chr5 | 12077607 | 12086144 | Cse_R012481 | Selenocysteine insertion sequence-binding protein 2-like | 8538 |
| chr5 | 12094349 | 12094978 | Cse_R012635 | -- | 630 |
| chr5 | 12095039 | 12095567 | Cse_R012683 | SHC-transforming protein 1 | 529 |
| chr5 | 12097398 | 12101746 | Cse_R012282 | Protein phosphatase 1G | 4349 |
| chr5 | 12104326 | 12105609 | Cse_R012306 | Protein MIS12 homolog | 1284 |
| chr5 | 12122172 | 12127908 | Cse_R012264 | Delta-like protein D | 5737 |
| chr5 | 12136522 | 12145348 | Cse_R012417 | Constitutive coactivator of peroxisome proliferator-activated receptor gamma | 8827 |
| chr5 | 12165021 | 12166251 | Cse_R012709 | -- | 1231 |
| chr5 | 12169214 | 12197527 | Cse_R012316 | RUN and FYVE domain-containing protein 2 | 28314 |
| chr5 | 12212784 | 12220578 | Cse_R012531 | SKI family transcriptional corepressor 1 homolog-B | 7795 |
| chr5 | 12243314 | 12259760 | Cse_R012293 | E3 SUMO-protein ligase PIAS1 | 16447 |
| chr5 | 12266840 | 12267826 | Cse_R012290 | Relaxin-3 receptor 1 | 987 |
| chr5 | 12272179 | 12280121 | Cse_R012318 | Mortality factor 4-like protein 1 | 7943 |
| chr5 | 12294899 | 12309373 | Cse_R012272 | Uveal autoantigen with coiled-coil domains and ankyrin repeats | 14475 |
| chr5 | 12664248 | 12667427 | Cse_R012416 | Ribonuclease T2 | 3180 |
| chr5 | 12670859 | 12698192 | Cse_R012341 | Ribosomal protein S6 kinase alpha-2 | 27334 |
| chr5 | 13646862 | 13647782 | Cse_R012750 | Protein phosphatase 1 regulatory subunit 3E | 921 |
| chr5 | 13649708 | 13652295 | Cse_R012622 | -- | 2588 |
| chr5 | 13655798 | 13661442 | Cse_R012256 | ATM interactor | 5645 |
| chr5 | 13663392 | 13666109 | Cse_R012619 | Centromere protein N | 2718 |
| chr5 | 13667272 | 13667688 | Cse_R012234 | Uncharacterized protein C16orf61 homolog | 417 |
| chr5 | 13674290 | 13676474 | Cse_R012557 | Chromodomain Y-like protein 2 | 2185 |
| chr5 | 13676483 | 13682225 | Cse_R012717 | Chromodomain Y-like protein 2 | 5743 |
| chr5 | 14272596 | 14275637 | Cse_R012576 | Forkhead box protein F1 | 3042 |
| chr5 | 14282689 | 14289892 | Cse_R012639 | Methenyltetrahydrofolate synthase domain-containing protein | 7204 |
| chr5 | 14297719 | 14299855 | Cse_R012747 | Dysbindin domain-containing protein 1 | 2137 |
| chr5 | 15646359 | 15668962 | Cse_R012527 | Signal peptide, CUB and EGF-like domain-containing protein 2 | 22604 |
| chr5 | 15671902 | 15673572 | Cse_R012726 | Transmembrane protein 9B | 1671 |
| chr5 | 15690775 | 15712939 | Cse_R012583 | Suppression of tumorigenicity 5 protein | 22165 |
| chr6 | 11525987 | 11533704 | Cse_R021264 | GTPase KRas | 7718 |
| chr6 | 11545146 | 11552018 | Cse_R021270 | Ras association domain-containing protein 8 | 6873 |
| chr6 | 11558801 | 11561529 | Cse_R021278 | Class E basic helix-loop-helix protein 41 | 2729 |
| chr6 | 11564442 | 11570139 | Cse_R021256 | Sarcospan | 5698 |
| chr6 | 11578746 | 11628080 | Cse_R021267 | Inositol 1,4,5-trisphosphate receptor type 2 | 49335 |
| chr6 | 11633027 | 11647409 | Cse_R021253 | Pleckstrin homology domain-containing family G member 7 | 14383 |
| chr6 | 11650028 | 11663976 | Cse_R021274 | Early endosome antigen 1 | 13949 |
| chr6 | 11668024 | 11672127 | Cse_R021255 | Diphosphoinositol polyphosphate phosphohydrolase 2 | 4104 |
| chr6 | 11676044 | 11677018 | Cse_R021272 | Ubiquitin-conjugating enzyme E2 N | 975 |
| chr6 | 11683029 | 11683976 | Cse_R021252 | Complement C1q tumor necrosis factor-related protein 4 | 948 |
| chr6 | 11686069 | 11696055 | Cse_R021269 | Bile acid receptor | 9987 |
| chr6 | 11701417 | 11709242 | Cse_R021254 | Vesicular glutamate transporter 3 | 7826 |
| chr6 | 11714116 | 11721684 | Cse_R021257 | Aryl hydrocarbon receptor nuclear translocator-like protein 2 | 7569 |
| chr6 | 11731527 | 11742484 | Cse_R021263 | Liprin-beta-1 | 10958 |
| chr6 | 15389219 | 15403398 | Cse_R013144 | Oxysterol-binding protein-related protein 5 | 14180 |
| chr6 | 15422383 | 15431865 | Cse_R013225 | Receptor-type tyrosine-protein phosphatase eta | 9483 |
| chr6 | 15455520 | 15461620 | Cse_R013069 | Tyrosine-protein phosphatase non-receptor type 5 | 6101 |
| chr6 | 15470184 | 15502143 | Cse_R012869 | Dual specificity protein phosphatase 8 | 31960 |
| chr6 | 15553991 | 15560560 | Cse_R013227 | MOB kinase activator 2 | 6570 |
| chr6 | 15563573 | 15564730 | Cse_R013129 | ATP-sensitive inward rectifier potassium channel 11 | 1158 |
| chr6 | 15565984 | 15567856 | Cse_R012967 | ATP-binding cassette sub-family C member 8 | 1873 |
| chr6 | 15572839 | 15574519 | Cse_R020847 | Tetratricopeptide repeat protein 38 | 1681 |
| chr6 | 15578397 | 15619943 | Cse_R020831 | Protein-methionine sulfoxide oxidase MICAL3 | 41547 |
| chr6 | 15915690 | 15922975 | Cse_R020836 | -- | 7286 |
| chr6 | 15948742 | 15952044 | Cse_R020845 | Teashirt homolog 3 | 3303 |
| chr6 | 16004943 | 16096680 | Cse_R020842 | Neural-cadherin | 91738 |
| chr7 | 2007100 | 2015436 | Cse_R020476 | Sodium-dependent multivitamin transporter | 8337 |
| chr7 | 2026149 | 2026421 | Cse_R020490 | Transcription factor 23 | 273 |
| chr7 | 2031022 | 2034116 | Cse_R020477 | Epoxide hydrolase 1 | 3095 |
| chr7 | 2037790 | 2038698 | Cse_R020488 | Signal recognition particle 9 kDa protein | 909 |
| chr8 | 9923835 | 9939361 | Cse_R014846 | Calpain-15 | 15527 |
| chr8 | 9945028 | 9945246 | Cse_R014833 | -- | 219 |
| chr8 | 9947466 | 9963709 | Cse_R014039 | Zinc finger MYND domain-containing protein 17 | 16244 |
| chr8 | 22250822 | 22365702 | Cse_R018228 | ELKS/Rab6-interacting/CAST family member 1 | 114881 |
| chr9 | 1320115 | 1329301 | Cse_R015764 | MICAL-like protein 2 | 9187 |
| chr9 | 1333422 | 1339609 | Cse_R015915 | Lysosomal alpha-glucosidase | 6188 |
| chrZ | 5644251 | 5645216 | Cse_R017298 | BCL2/adenovirus E1B 19 kDa protein-interacting protein 3-like | 966 |
| chrZ | 5648308 | 5656586 | Cse_R016719 | Dihydropyrimidinase-related protein 2 | 8279 |
| chrZ | 5649880 | 5650824 | Cse_R022131 | -- | 945 |
| chrZ | 5662390 | 5666295 | Cse_R017033 | Alpha-1A adrenergic receptor | 3906 |
| chrZ | 5673852 | 5675869 | Cse_R017053 | UPF0414 transmembrane protein C20orf30 homolog | 2018 |

**Table S6. Candidate regions that harbors significantly associated SNPs (P < 10^-6^).**

| **Chromosome** | **Start** | **End** | **Length (bp)** | **No. of GWAS-SNPs** |
| --- | --- | --- | --- | --- |
| chr5 | 4357294 | 4680000 | 322706 | 2 |
| chr5 | 8436346 | 8460000 | 23654 | 1 |
| chr5 | 10800001 | 11096344 | 296343 | 3 |
| chr5 | 15660001 | 15696676 | 36675 | 1 |
| chr12 | 15280001 | 15309909 | 29908 | 1 |
| chr17 | 12991022 | 13031079 | 40057 | 1 |
| Total | NA | NA | 749343 | 9 |

**Table S7. The genes located in the candidate regions that contain GWAS signals.**

| **CHR** | **Start of gene** | **End of gene** | **GeneID** | **Annotation** | **Gene length** |
| --- | --- | --- | --- | --- | --- |
| chr5 | 4359508 | 4362376 | Cse_R012249 | Ataxin-1-like | 2868 |
| chr5 | 4371138 | 4373551 | Cse_R012304 | Zinc finger protein 821 | 2413 |
| chr5 | 4387543 | 4395898 | Cse_R012323 | AP-1 complex subunit gamma-1 | 8355 |
| chr5 | 4403577 | 4417838 | Cse_R012325 | PH domain leucine-rich repeat-containing protein phosphatase 2 | 14261 |
| chr5 | 4423430 | 4425086 | Cse_R012263 | MARVEL domain-containing protein 3 | 1656 |
| chr5 | 4430531 | 4440230 | Cse_R012550 | Ubiquitin carboxyl-terminal hydrolase 10 | 9699 |
| chr5 | 4448247 | 4457675 | Cse_R012549 | Cysteine-rich secretory protein LCCL domain-containing 2 | 9428 |
| chr5 | 8459433 | 8501872 | Cse_R012380 | Protein KIAA1199 | 42439 |
| chr5 | 10822411 | 10823186 | Cse_R017645 | Retinoblastoma-like protein 1 | 775 |
| chr5 | 10955212 | 10963719 | Cse_R017647 | TOX high mobility group box family member 3 | 8507 |
| chr5 | 15671902 | 15673572 | Cse_R012726 | Transmembrane protein 9B | 1670 |
| chr5 | 15690775 | 15712939 | Cse_R012583 | Suppression of tumorigenicity 5 protein | 22164 |
| chr12 | 13200187 | 13211170 | Cse_R021485 | Fibroblast growth factor receptor 2 | 10983 |
| chr12 | 15268542 | 15289434 | Cse_R004079 | WD repeat-containing protein 96 | 20892 |
| chr12 | 15289567 | 15290322 | Cse_R004022 | Swi5-dependent recombination DNA repair protein 1 homolog | 755 |
| chr12 | 15305384 | 15327097 | Cse_R004332 | Collagen alpha-1(XVII) chain | 21713 |
| chr17 | 12994163 | 12996177 | Cse_R008084 | THO complex subunit 6 homolog | 2014 |
| chr17 | 12996646 | 12998847 | Cse_R007898 | Mediator of RNA polymerase II transcription subunit 15 | 2201 |
| chr17 | 12998794 | 13004280 | Cse_R008461 | Mediator of RNA polymerase II transcription subunit 15 | 5486 |
| chr17 | 13007294 | 13010930 | Cse_R008453 | F-box/LRR-repeat protein 19 | 3636 |
| chr17 | 13012576 | 13016010 | Cse_R008168 | NEDD8-conjugating enzyme Ubc12 | 3434 |
| chr17 | 13017961 | 13022325 | Cse_R008006 | E3 ubiquitin/ISG15 ligase TRIM25 | 4364 |
| chr17 | 13026716 | 13032059 | Cse_R007599 | Rho GTPase-activating protein 27 | 5343 |

**Table S8. The SNPs associated with *fgfr2* and *pkca* gene.**

| **SNP ID** | **Chr** | **Position** | **Ref** | **Alt** | **-log**_10_ *P* | **Peak effect** | **Gene ID** | **Gene annotation** |
| --- | --- | --- | --- | --- | --- | --- | --- | --- |
| CsSNP10 | chr12 | 13200933 | A | G | 6.36 | Intronic | *fgfr2* | Fibroblast growth factor receptor 2 |
| CsSNP29 | chr17 | 8095454 | C | T | 6.08 | intronic | *pkca* | Protein kinase C alpha type |
